# The biofilm inhibitor Carolacton inhibits planktonic growth of virulent pneumococci via a conserved target

**DOI:** 10.1038/srep29677

**Published:** 2016-07-11

**Authors:** Jannik Donner, Michael Reck, Simone Bergmann, Andreas Kirschning, Rolf Müller, Irene Wagner-Döbler

**Affiliations:** 1 Department of Medical Microbiology, Group Microbial Communication, Helmholtz Centre for Infection Research, Braunschweig, Germany; 2 Institute of Microbiology, Technische Universität Braunschweig, Braunschweig, Germany; 3 Institute of Organic Chemistry and Center of Biomolecular Drug Research (BMWZ), Leibniz Universität Hannover, Hannover, Germany; 4 Department of Microbial Natural Products, Helmholtz Institute for Pharmaceutical Research Saarland (HIPS), Helmholtz Centre for Infection Research and Pharmaceutical Biotechnology, Saarland University, Saarbrücken, Germany

## Abstract

New antibacterial compounds, preferentially exploiting novel cellular targets, are urgently needed to fight the increasing resistance of pathogens against conventional antibiotics. Here we demonstrate that Carolacton, a myxobacterial secondary metabolite previously shown to damage *Streptococcus mutans* biofilms, inhibits planktonic growth of *Streptococcus pneumoniae* TIGR4 and multidrug-resistant clinical isolates of serotype 19A at nanomolar concentrations. A Carolacton diastereomer is inactive in both streptococci, indicating a highly specific interaction with a conserved cellular target. *S. mutans* requires the eukaryotic-like serine/threonine protein kinase PknB and the cysteine metabolism regulator CysR for susceptibility to Carolacton, whereas their homologues are not needed in *S. pneumoniae*, suggesting a specific function for *S. mutans* biofilms only. A bactericidal effect of Carolacton was observed for *S. pneumoniae* TIGR4, with a reduction of cell numbers by 3 log units. The clinical pneumonia isolate Sp49 showed immediate growth arrest and cell lysis, suggesting a bacteriolytic effect of Carolacton. Carolacton treatment caused a reduction in membrane potential, but not membrane integrity, and transcriptome analysis revealed compensatory reactions of the cell. Our data show that Carolacton might have potential for treating pneumococcal infections.

The resistance of pathogens against antibiotics is increasing worldwide^1^, while the number of new antibiotics in the clinical pipeline remains to be low^2^. As antibiotic treatment often fails against pathogens that have developed resistance, it disturbs the human microbiome at the same time^3^, which has become a major concern. New antibacterial compounds are urgently needed, preferably compounds that act on novel or underexploited cellular mechanisms and could be used as a starting point for rational drug design^4^.

Carolacton is a secondary metabolite produced by the myxobacterium *Sorangium cellulosum.* It is a macrolide ketocarbonic acid^5^ that can be synthesised *de novo*^6^,^7^. Carolacton was discovered to cause membrane damage of biofilms of the caries pathogen *Streptococcus mutans* at nanomolar concentrations and is non-toxic to eukaryotic cells^8^. Dental composite materials incorporating Carolacton maintain a strong *in vitro* activity against *S. mutans* biofilms while the mechanical properties of the material are not affected^9^, suggesting that such composites might be applicable to reduce secondary caries formation. Carolacton has only a weak effect on the growth of *S. mutans*^10^, but inhibits growth of *Streptococcus oralis*^11^ and also of *Escherichia coli* TolC with a minimal inhibitory concentration (MIC) as low as 0.06 μg/ml^5^. Carolacton-treated cultures show increased septum formation, elongated cells, and cell chains, suggesting defects in cell division or cell wall synthesis^8^,^10^,^11^,^12^. All chemical modifications of Carolacton tested until now rendered the molecule inactive^11^. Based on these studies, we hypothesised that the primary effect of Carolacton is a disturbance of cell division or cell wall synthesis through a stereospecific interaction with a conserved cellular target. Moreover, we hypothesised that the death of *S. mutans* biofilm cells, which occurs several hours after Carolacton treatment at low pH only^10^, is a secondary effect that might be unique to this species due to the strong acidification of mutans streptococci within mature biofilms^13^.

To investigate these hypotheses and to understand the mode of action of Carolacton in more detail, we studied the response of *S. pneumoniae* to Carolacton and compared it with that of *S. mutans*. *S. pneumoniae* is a ubiquitous colonizer of the nasopharynx^14^ whose carriers usually remain asymptomatic. Nevertheless, invasive and non-invasive pneumococcal infections account for almost half a million hospitalizations and approximately 22.000 deaths in the United States annually^15^. A continuous increase in multidrug resistance (MDR, resistant to ≥3 classes of antimicrobials) is being reported^16^. Particularly the prevalence of *S. pneumoniae* strains of serotype 19A has increased drastically from 5% in the mid-1990’s to up to 48% in 2008^17^. Serotype 19A isolates have the highest proportion of MDR and extensively drug resistant strains (XDR, resistant to ≥5 classes of antimicrobials)^18^. Consequently, the search for novel antimicrobial compounds and novel cellular targets remains of crucial importance, especially with regard to pneumococci.

In this study, we focused on *S. pneumoniae* TIGR4 as an extensively characterized strain^19^. For the first time, we report complete growth inhibition of planktonically growing cells. We investigated growth inhibition by Carolacton of MDR and XDR clinical isolates of *S. pneumoniae* classified as serotype 19A. To clarify if *S. pneumoniae* shares the molecular target of Carolacton with *S. mutans*, we tested its sensitivity against 9-*epi*-Carolacton. This Carolacton derivative has the opposite absolute configuration at C-9, the area of macrolactone formation, and is inactive against *S. mutans*^11^. Cell death of *S. mutans* biofilm cells has been shown to be dependent on the eukaryotic-like serine/threonine protein kinase (eSTK) PknB^10^ and the regulator of cysteine metabolism CysR^20^. Therefore, their homologues in *S. pneumoniae* TIGR4 were deleted and their response to Carolacton determined. Finally we investigated the transcriptome, including small regulatory RNAs, of Carolacton-treated cultures of *S. pneumoniae* TIGR4, constructed knock-out mutants of strongly differentially expressed genes and tested their susceptibility. Our data demonstrate that Carolacton acts via the same molecular target in *S. mutans* and *S. pneumoniae.* Death of biofilm cells of *S. mutans* is a late secondary effect. The fast (within 5 min) and distinct transcriptional response to Carolacton treatment represents a compensatory mechanism, since deletion of those genes does not render the mutants insensitive to Carolacton. Finally, the inhibition of growth of *S. pneumoniae* by Carolacton shows the characteristics of bactericidal and in some cases even bacteriolytic antibiotics.

## Results

### Carolacton inhibits growth of *S. pneumoniae* TIGR4 in planktonic culture

**Figure 1:**
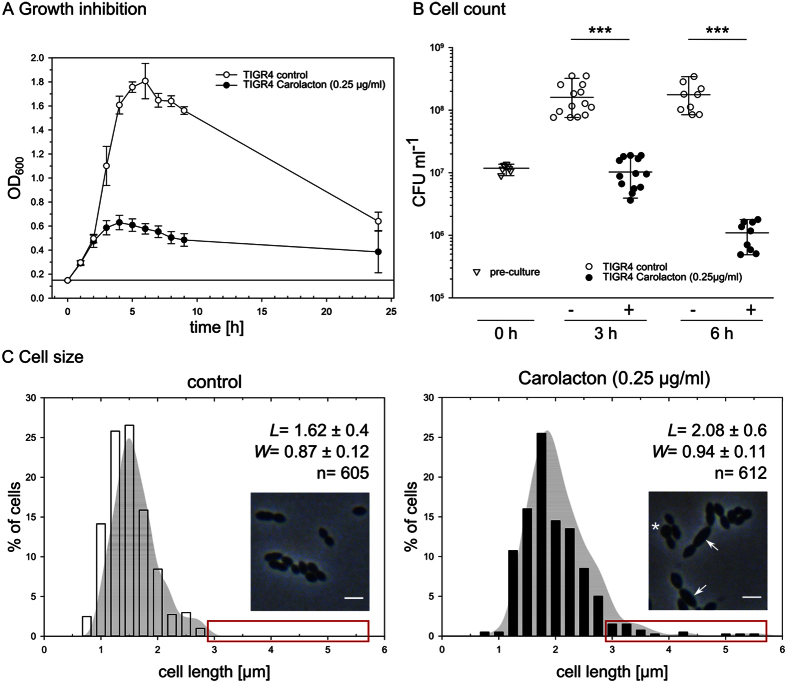
Treatment with Carolacton inhibits growth of *S. pneumoniae* TIGR4 and affects cell count and cell size. **(A)** The growth curve indicates a growth inhibition of planktonically growing cells of *S. pneumoniae* TIGR4 by Carolacton at a final concentration of 0.25 μg/ml. The start OD_600_ is indicated by a solid horizontal line. Depicted is the average inhibition from three independent experiments (±standard deviation). **(B)** Determination of viable cell numbers by plating for colony counts. The cell number was determined after plating serial dilutions and counting the CFU. (+) 0.25 μg/ml Carolacton and (−) control. Data are representative for at least three biologically independent experiments (±s.d.). *P* value: *** = < 0.001; Mann-Whitney U. **(C)** Cell length distribution of cells grown with 0.25 μg/ml Carolacton for 3 h. A length of ≥ 3 μm was only observed for treated cells (red box). In the microscope image, elongated cells are indicated by arrows, a cell showing a bulky and voluminous morphology is marked by an asterisk (*). The average cell length (*L*) and width (*W*) with standard deviations are shown for n cells from three independent experiments. Scale bar: 2.5 μm. Changes in *L* and *W* were statistically significant for *P* < 0.05 as determined by non-parametric Mann-Whitney-U test; *P*(*L*) < 0.001, *P*(*W*) = 0.011.

Carolacton (0.25 μg/ml final concentration) was added to cultures of *S. pneumoniae* TIGR4 prior to the onset of the exponential phase (OD_600_ = 0.15, termed “t_0_”). Carolacton-treated cultures grew at the same rate as untreated controls for about 2 h, after which the OD remained constant or decreased (Fig. 1A), suggesting a bactericidal activity^21^. The Carolacton-treated cultures typically reached a maximal OD_600_ of 0.55–0.60, while the controls grew up to an OD_600_ of 1.8. A maximal inhibition of ~67% could be observed after 9 h of growth. Since *S. pneumoniae* autolyzes after reaching the stationary phase^22^, final cell densities after 24 h were very low and almost the same in treated and untreated cultures. The number of viable cells was determined by counting colony forming units (CFUs). Figure 1B shows that after 3 h of Carolacton treatment, the number of CFUs was reduced by 1 log unit (92.4%) and decreased further by more than 2 log units (99.4%) after 6 h. Since this decrease in CFUs was not accompanied by a reduction in OD_600_, the data indicate reduced cell viability in the presence of Carolacton. For *S. pneumoniae* TIGR4, the minimal inhibitory concentration (MIC) of Carolacton was 0.06 μg/ml (Table 1). Cells grown with Carolacton showed a more voluminous phenotype and a fraction of the cells showed a moderate but significant increase in size (Fig. 1C). On average, cell length increased from 1.62 ± 0.4 μm to 2.08 ± 0.6 μm, while cell width increased from 0.87 ± 0.12 μm to 0.94 ± 0.11 μm. Even a 100-fold increase (0.025–2.5 μg/ml) of the Carolacton concentration did not increase the inhibition of growth considerably (Supplementary Fig. S1). This is in full accordance with Kunze *et al*. who reported a concentration-independent effect of Carolacton on death of *S. mutans* biofilms over the same concentration range^8^.

### Carolacton inhibits planktonic growth of *S. pneumoniae* serotype 19A clinical isolates

**Figure 2:**
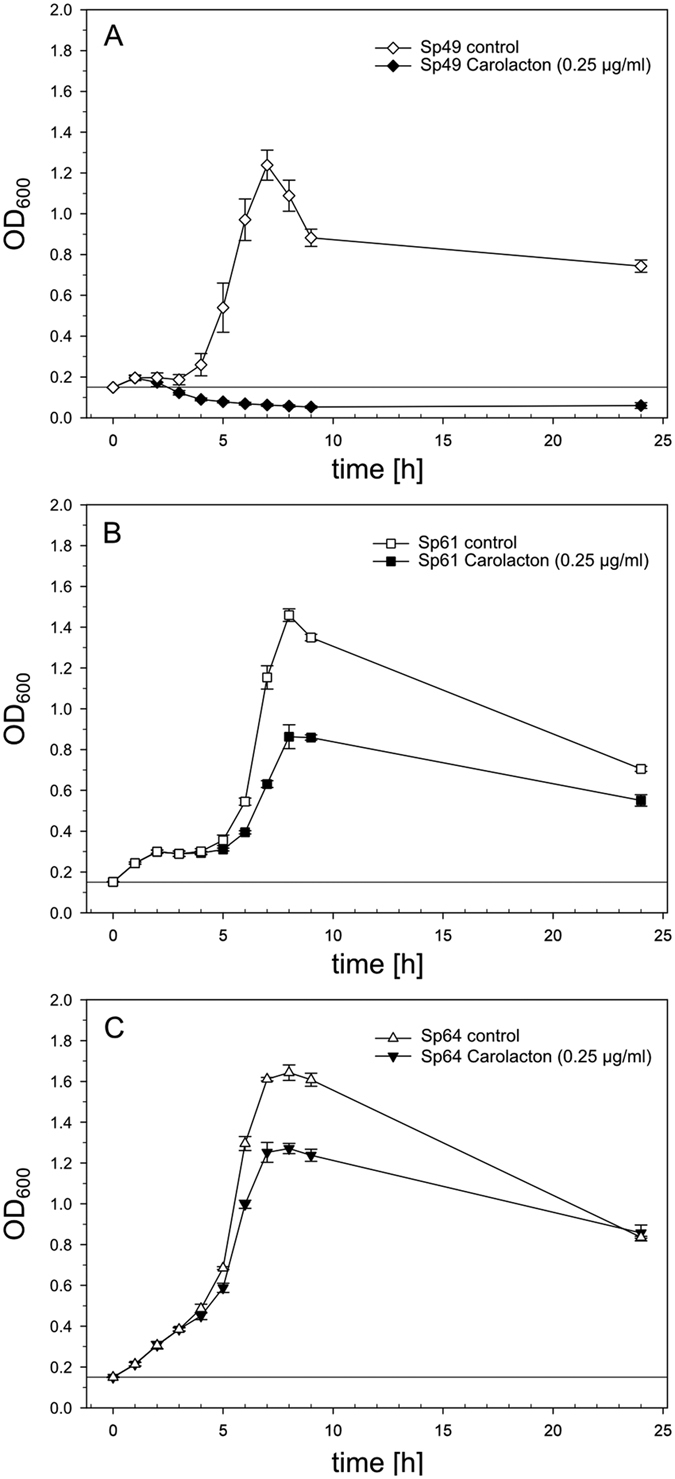
Activity of Carolacton against *S. pneumoniae* clinical isolates of serotype 19A. Growth inhibition of pneumococcal clinical isolates Sp49 (pneumonia/sepsis isolate, (**A**)), Sp61 (pleuritis isolate, (**B**)) and Sp64 (mid ear isolate, (**C**)) in THBY. Carolacton was added at a final concentration of 0.25 μg/ml prior to the onset of exponential growth at an OD_600_ of 0.15. The start OD_600_ is indicated by a solid horizontal line. The growth curves show the average inhibition calculated from three independent experiments (±s.d.).

To investigate the effectiveness of Carolacton treatment on *S. pneumoniae* strains found in pneumococcal disease, three different recent clinical isolates of *S. pneumoniae*, representing drug resistant serotype 19A strains (Table 2, Supplementary Table S1), were subjected to Carolacton susceptibility testing. Isolate Sp49 had been obtained from the blood of a septic pneumonia patient and was resistant against two classes of antibiotics. Isolate Sp61 and Sp64 were XDR (resistant to ≥5 classes of antibiotics); they were isolated from the thorax drainage of a pleuritis patient and by swab from an ear canal of a non-symptomatic patient, respectively. The growth of all three clinical isolates was inhibited by Carolacton, but to a widely varying extent (Fig. 2). The clinical pneumonia isolate (Sp49) was most sensitive and showed a different behaviour from TIGR4: it started to die immediately and after 2 h its OD was lower than at t_0_, suggesting a bacteriolytic effect of Carolacton (Fig. 2A). A maximum inhibition of 94.9% could be observed after approximately 6–7 h of growth, this was the same time point at which the control culture of Sp49 typically reached its peak (OD_600_ ~1.2). For the clinical pleuritis isolate (Sp61) and the non-invasive mid-ear serotype 19A isolate (Sp64), the period of uninhibited growth was longer than for the TIGR4 strain, and they reached higher final culture densities than TIGR4. The pleuritis isolate Sp61 showed a maximal inhibition of growth of about 45% after 6 h (Fig. 2B). For the non-invasive mid-ear isolate Sp64, the maximal inhibition was less than that detected for the pleuritic isolate, reaching a maximum inhibition of 23%. Moreover, the inhibition peak was retarded and observed after 8 h (Fig. 2C). Carolacton had a strong lytic effect on clinical isolate Sp49; the MIC value determined for this isolate was ≤0.03 μg/ml and thus lower than for TIGR4. Drug-resistant isolates Sp61 and Sp64, in contrast, were highly tolerant of Carolacton and were only inhibited at 64 μg/ml Carolacton (Table 1). These results indicate that Carolacton inhibits growth of clinical *S. pneumoniae* isolates with varying efficiency. The degree of inhibition was not affected by the change of serotype from TIGR4 (serotype 4) to the clinical isolates (serotype 19A). Interestingly, growth inhibition was weaker in the pleuritis isolate Sp61 and in the non-invasive isolate Sp64, which elicit multiple resistances to antibiotics compared to the pneumonia isolate.

### The interaction of Carolacton with its cellular target is highly stereo-specific

**Figure 3:**
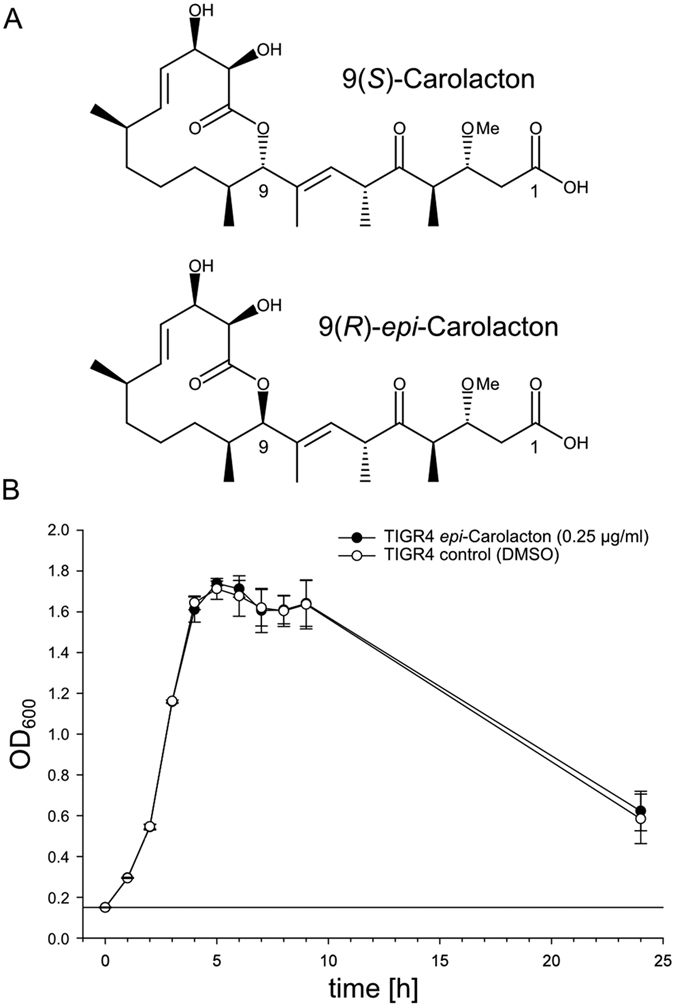
Effect of *epi*-Carolacton on growth of *S. pneumoniae* TIGR4. **(A)** Chemical structures of native 9(*S*)-Carolacton (top) and the 9(*R*)-epimer (*epi*-Carolacton, bottom) obtained by total synthesis. **(B)** Cells were treated with *epi*-Carolacton (black dots) at a final concentration of 0.25 μg/ml at an OD_600_ of 0.15. As control, pneumococci were cultivated with an equivalent volume of solvent (DMSO, white dots). The experiment was repeated independently at least three times.

The Carolacton derivative 9(*R*)-Carolacton (*epi*-Carolacton) is a diastereomer of Carolacton exhibiting an inversion at C–9 [(*S*)→(*R*)] (Fig. 3A). Although the structural changes are minute, *epi*-Carolacton is biologically inactive against *S. mutans* in biofilms^11^. Likewise, 0.25 μg/ml *epi*-Carolacton, a concentration which is generally applied in our growth experiments with streptococci, did not inhibit growth of *S. pneumoniae* TIGR4 (Fig. 3B). Thus the interaction between Carolacton and its molecular target must be highly specific with respect to the stereochemistry at position 9 of the molecule, heavily influencing the topology of the macrocycle. Due to the stereospecificity of this interaction we suggest that the cellular target of Carolacton is likely to be identical between *S. mutans* and *S. pneumoniae*, in spite of the differences of their physiological responses.

### The serine/threonine protein kinase StkP and the regulator CysR are not required for growth inhibition by Carolacton

**Figure 4:**
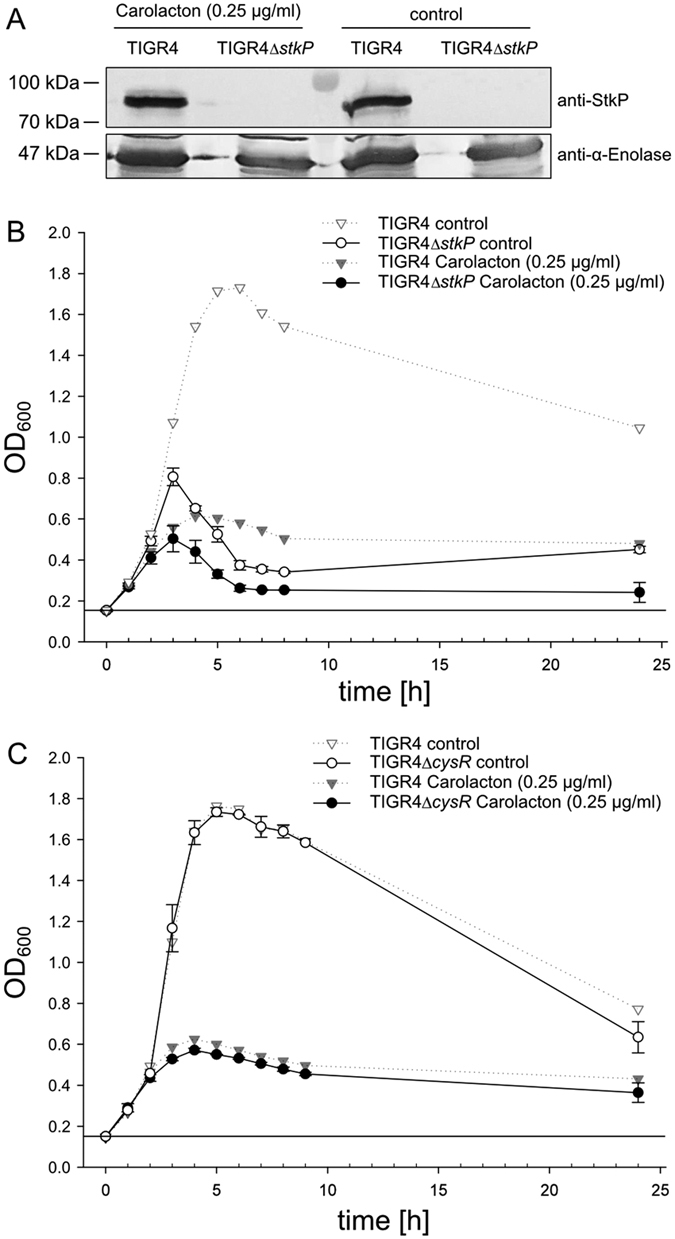
Growth inhibition of *S. pneumoniae* TIGR4Δ*stkP* and TIGR4Δ*cysR* by treatment with Carolacton. **(A)** Immunoblot analysis with StkP-specific antibodies of *S. pneumoniae* TIGR4 and TIGR4Δ*stkP* confirms absence of StkP-expression in the TIGR4Δ*stkP* deletion mutant. Pneumococcal α-enolase was detected with specific antibodies as loading control. Presented are two parts of the same blot treated with different primary antibodies. **(B)** Growth inhibition of a TIGR4Δ*stkP* mutant (solid lines) is plotted after addition of 0.25 μg/ml Carolacton (black dots) and without Carolacton as control (white dots). **(C)** Inhibition of a planktonically growing TIGR4Δ*cysR* mutant with (black dots) and without (white dots) Carolacton. Inhibition of the TIGR4 wild type strain (dotted lines), used as controls during both assays (Carolacton-treated = black triangles, control = white triangles), is displayed as a semi-transparent plot in **(A)** and **(B)**. All growth curves present the average of three independent biological replicates.

Gene deletion of the signalling kinase PknB induces Carolacton resistance of *S. mutans* biofilms^10^. The *S. pneumoniae* genome encodes *stkP* (*sp_1732*) as corresponding homologue to *S. mutans pknB*. Therefore, the role of the homologue of PknB in *S. pneumoniae*, StkP, in Carolacton susceptibility of *S. pneumoniae* TIGR4 was tested. Since the molecular target of Carolacton appears to be highly conserved in those two organisms, this analysis will clarify whether a bacterial eukaryotic-like serine/threonine protein kinase (eSTK) is the direct molecular target of Carolacton. Due to the strong indications of eSTKs as the primary target of Carolacton^10^, in addition to PCR amplification and DNA-sequencing, the absence of the StkP protein in theΔ*stkP* isogenic mutant was confirmed by immunoblotting using polyclonal anti-StkP antibodies^23^ (Fig. 4A). The StkP deletion strain showed formation of elongated cells and multiple unconstricted septa, as reported previously^23^. Its growth was highly impaired; nevertheless, Carolacton treatment led to growth inhibition (Fig. 4B). The data show that the Δ*stkP* TIGR4 strain is susceptible to Carolacton, which is in sharp contrast to the findings described for *S. mutans*^10^.

Deletion of the cysteine metabolism regulator CysR also resulted in a Carolacton-insensitive mutant in *S. mutans*^20^. The CysR homologue (SP_0927) was deleted in *S. pneumoniae* and tested for susceptibility to Carolacton. Similar to the Δ*stkP* strain, growth of the Δ*cysR* mutant was strongly inhibited by Carolacton (Fig. 4C). Thus, the pneumococcal homologues to both proteins mediating the susceptibility to Carolacton in *S. mutans*, StkP and CysR, are most likely not directly involved in the Carolacton response in *S. pneumoniae*, in spite of the presumably conserved molecular target of Carolacton in those two species. We conclude that PknB and CysR are involved in the mechanisms of membrane damage at low pH in *S. mutans*, which is a late secondary response to Carolacton treatment. In order to confirm this hypothesis, the effect of Carolacton on membrane integrity and membrane potential of *S. pneumoniae* TIGR4 was analysed.

### Effect of Carolacton on cytoplasmic membrane integrity and membrane potential in *S. pneumoniae*

**Figure 5:**
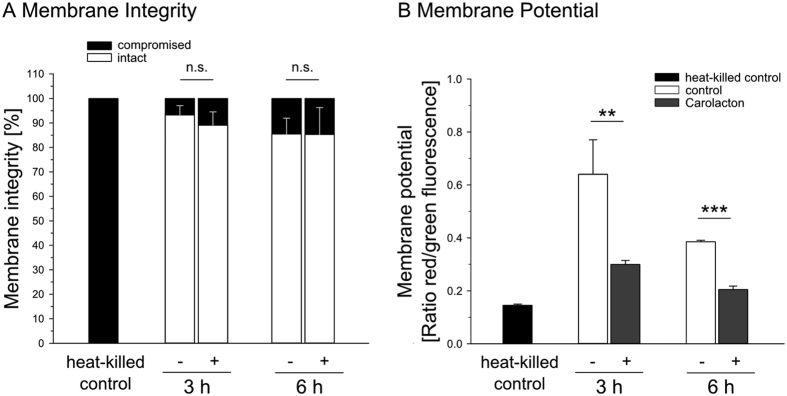
Effect of Carolacton-treatment on the membrane integrity and membrane potential of *S. pneumoniae* TIGR4. **(A)** Cells grown with 0.25 μg/ml Carolacton (+) and controls (−) were stained with Syto 9/PI and analysed using flow cytometry as exemplified in Fig. S8. Error bars of stained heat-killed controls (red bars) are not visible, as continuously more than 99.9% of all heat-treated cells were killed and emitted red fluorescence. **(B)** Changes in membrane potential were quantified by flow cytometric analysis of DiOC_2_(3)-stained planktonic cells. The relative membrane potential was calculated ratiometrically. Analyses were carried out with 30 μM DiOC_2_(3). Heat-killed cells served as a control for fully depolarized cells. The bar charts present the average of three biological replicates (±s.d.). *P* value: n.s. = > 0.05; ** = < 0.01; *** = < 0.001; two-tailed Student’s t-test.

For *S. mutans* it has been reported that Carolacton leads to death of biofilm cells at low pH by disturbing the integrity of the plasma membrane and thereby causing leakage of cytoplasmic proteins and DNA^10^. Membrane damage of *S. pneumoniae* cells was similarly determined by LIVE/DEAD staining during exponential and stationary phase, 3 h and 6 h after addition of Carolacton, respectively. Heat-killed *S. pneumoniae* cells were included as a control and a calibration curve was recorded using flow cytometry (Supplementary Fig. S2). No indication of *S. pneumoniae* cell death by Carolacton treatment was detected (Supplementary Fig. S3). Controls and Carolacton-treated cultures both contained a maximum of 14% cells with defective plasma membranes (Fig. 5A).

We then used staining with the fluorescent dye *3*,*3*-Diethyloxacarbocyanine iodide (DiOC_2_(3)) to determine changes in the MP upon Carolacton treatment. Since the measured fluorescence intensities are directly influenced by the size of the stained particles, and Carolacton treatment affects the cell size, a size-independent ratiometric technique was applied^24^. Carolacton treatment caused a time-dependent depolarization of the membrane potential (Fig. 5B, Supplementary Fig. S4). Approximately 48% of the cells showed a membrane depolarization, indicated by a reduction of the red/green fluorescent ratio, after 3 h. Membrane depolarization continued during the next 3 h, though not as fast as during the first time interval. After 6 h, the red/green fluorescent ratio dropped to ~34% of that measured for an exponentially growing control after 3 h, and was reduced to ~56% when compared to the control at the same time point. These results indicate a Carolacton-dependent disturbance of the membrane potential during growth that is not the result of a porous plasma membrane.

### Transcriptional response of *S. pneumoniae* TIGR4 to Carolacton

We analysed the transcriptome of *S. pneumoniae* TIGR4 cells growing with Carolacton starting 5 min after addition of the drug, until 3 h after Carolacton addition. Total RNA was fractionated into two size classes: >200 nt and ≤200 nt, and differential regulation of mRNAs (30–4776 nt), small RNAs (37–486 nt) and tRNAs (71–90 nt) was assessed individually. We noted a high number of reads for the previously identified housekeeping sRNAs 6S RNA (194 nt)^25^ and RNase P (391 nt)/tmRNA (348 nt)^26^,^27^ in the samples containing small transcripts (≤200 nt) or in the samples containing large transcripts (>200 nt), respectively, confirming the applicability of our sequencing approach for reliable separation of transcripts according to length (Supplementary Dataset 1). Since differential transcription after Carolacton treatment was exclusively limited to the later time points (t_120_/t_180_), we chose to only sequence three time points (t_0_, t_5_ and t_180_) for analysis of short small RNAs/tRNAs. The RNA-seq data of biological replicates showed a very high Pearson correlation between 0.93 for small regulatory RNAs/tRNAs and 0.99 for large regulatory RNAs/mRNAs.

None of the 58 tRNAs of *S. pneumoniae* TIGR4 were significantly differentially expressed after Carolacton treatment (Supplementary Fig. S5, Supplementary Dataset 1).

### Differential transcription of small regulatory RNAs

A total number of 181 known small sRNAs (≤200 nt) and 21 known long sRNAs (>200 nt) were analysed. Transcription of the vast majority of sRNAs was confirmed by obtaining high read counts after mapping (Supplementary Dataset 1). Seven small sRNAs were differentially transcribed at t_180_ only (Supplementary Fig. S5). The highest upregulated small sRNA *trn0057* (log_2_FC~3.9) is located downstream of the *sp_0119-sp_0120* operon and its function is unknown. Ten long sRNAs were differentially regulated at t_120_ and t_180_ (Supplementary Fig. S5) of which only two (R12 and F20) have been investigated and were shown to be important for virulence in mice^28^.

### Differential transcription of mRNAs

RNA-seq data was analysed for differential expression of 1985 large (>200 nt) and 120 small (≤200 nt) transcripts of *S. pneumoniae* TIGR4, accounting for 2105 protein-coding genes (Supplementary Dataset 2). Carolacton caused a maximum of differential gene expression after 120 and 180 min. Overall, only 1.9% of the 2105 protein encoding genes were differentially transcribed (log_2_FC ≥ ± 2, FDR < 0.01) (Supplementary Fig. S6).

**Figure 6:**
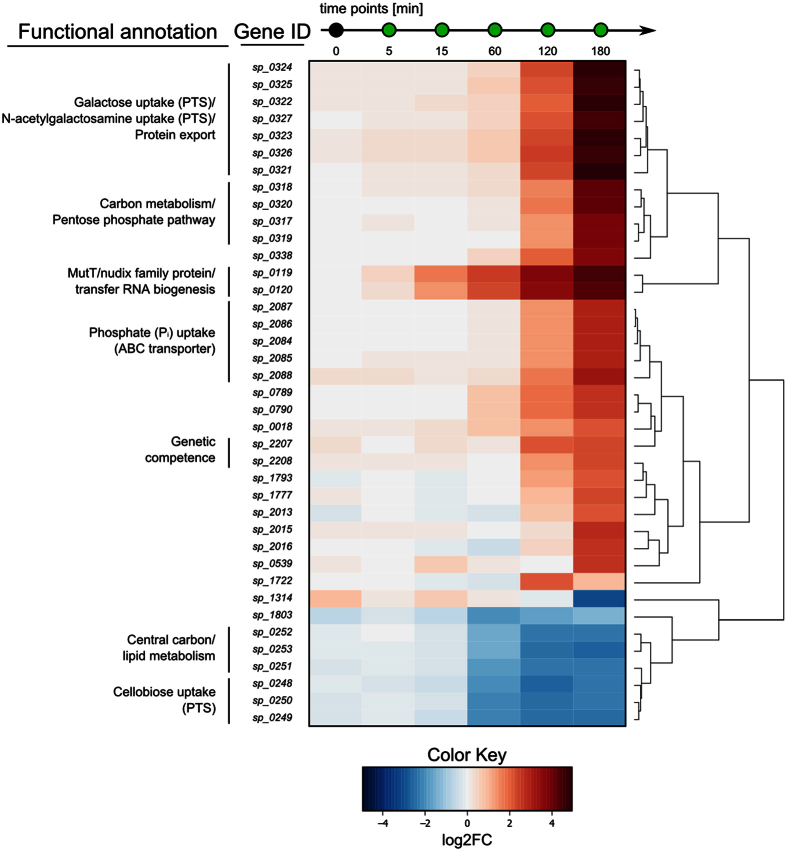
Differentially regulated mRNAs in *S. pneumoniae* TIGR4 upon treatment with Carolacton. The heat map illustrates the 39 most differentially expressed genes (mRNA > 200 nt) after addition of Carolacton (0.25 μg/ml). The cut-off for differentially regulated genes was set at log_2_FC ≥ ± 2 and FDR < 0.01 for at least one sample during the time course. RNA-seq data was analysed using Rockhopper and the edgeR package for R. The heat map was constructed using the R package gplots. Main operon structures are indicated by vertical lines on the left. Information on encoded proteins and their involvement in biological pathways were obtained from KEGG ORTHOLOGY or KEGG BRITE databases.

Two transcripts smaller than 200 nt were differentially regulated, a type II DNA modification methyltransferase (*sp_0569*) and the 50S ribosomal protein L28 (*sp_0441*) (Supplementary Fig. S5). 39 transcripts larger than 200 nt were strongly differentially expressed (log_2_FC ≥ ± 2 and FDR < 0.01 for at least one time point) (Fig. 6), 26 of which are encoded within 7 operons on the TIGR4 chromosome (Table 3). Differential expression of *sp_0119* and *sp_0120* was detected already 5 min after addition of Carolacton and continued to increase during the experiment to a final log_2_FC of 4.4 and 4.3 after 180 min, respectively. *Sp_0119* codes for a MutT/Nudix family protein and *sp_0120* encodes the glucose-inhibited division protein A (GidA).

### Influence of *sp_0119* and *sp_0120* on susceptibility of *S. pneumoniae* TIGR4 to Carolacton

Since the gene products of both *sp_0119* and *sp_0120* are involved in central cellular processes we wondered whether an uncontrolled transcriptional upregulation of this operon could cause the growth inhibitory effect of Carolacton. Therefore, single deletion mutants of *sp_0119* and *sp_0120* and a Δ*sp_0119-sp_0120* double mutant were constructed in *S. pneumoniae* TIGR4. Growth of all three mutant strains was still significantly inhibited by Carolacton treatment (Supplementary Fig. S7).

### Comparison of the transcriptome of *S. mutans* UA159 and *S. pneumoniae* TIGR4 in response to Carolacton

Until now, the only other organism for which the influence of Carolacton has been studied in more detail is the human oral pathogen *S. mutans*. Therefore, the RNA-seq data obtained in this study were compared to microarray data previously obtained for Carolacton-treated *S. mutans* biofilm cells^10^. 22.8% of all genes were differentially transcribed at 180 min in *S. pneumoniae* at a log_2_FC of ≥ ± 0.8 (Supplementary Fig. S6), which had previously been used for *S. mutans*^10^. At this cut-off, the effect of Carolacton was highly pleiotropic. Supplementary Fig. S8 shows a direct comparison between log_2_FC of the most strongly differentially expressed genes in *S. mutans* compared to their homologues in *S. pneumoniae*. Overall, the patterns of transcriptional changes were similar for some, but not all genes, and the timing and extent of the responses shows differences (Supplementary Fig. S8). For example, upregulation of the ComDE two-component system (TCS) for genetic competence is observed in both species, but stronger and earlier in *S. pneumoniae*, and might represent a general stress response^29^. The most conspicuous transcriptional response in *S. mutans* was the instantaneous strong upregulation of genes for pyrimidine biosynthesis. In *S. pneumoniae*, transcription of these genes showed a different pattern, indicating that their regulation in *S. mutans* might be related to the membrane damage occurring at later stages of biofilm growth. In *S. mutans*, downregulation of sortase A was observed. In *S. pneumoniae* TIGR4, neither sortase A (*srtA*, *sp_1218*) nor its three sortase paralogs (*srtBCD, sp_0466-sp_0468*)^30^ were differentially transcribed. Also in contrast to *S. mutans*, the VicKRX TCS was not differentially expressed upon Carolacton treatment in *S. pneumoniae*. Consequently, the VicKRX regulon also showed a different pattern: only transcripts of the cell shape proteins MreC and MreD were upregulated, although earlier and stronger than in *S. mutans*. In *S. mutans*, cell wall metabolism-related genes were upregulated, while the respective homologues in *S. pneumoniae* TIGR4 were not significantly differentially expressed.

Interestingly, the *gidA* homologue of *S. mutans* (*smu_2141*) was among the most strongly upregulated transcripts like in *S. pneumoniae*, although its upregulation was weaker and occurred later than in the pneumococcus (Supplementary Fig. S8).

Taken together, the transcriptional response of *S. mutans* and *S. pneumoniae* to Carolacton appears to be a pleiotropic stress response, different between species, and not a specific and focused transcriptional response as assumed after studies with *S. mutans* before^10^.

## Discussion

For the first time we report on the physiological effect of Carolacton on the clinically relevant human pathogen *S. pneumoniae.* Growth of planktonic cultures of *S. pneumoniae* is already inhibited by Carolacton at a concentration as low as 0.025 μg/ml (Supplementary Fig. S1). Growth inhibition starts about 2 h after application of the drug; at later time points, the optical density remains constant, but the number of viable cells continues to decrease. Such behaviour is typically observed when treating pneumococci with bactericidal antibiotics^21^. Growth inhibition by Carolacton is concentration-independent, which could indicate that the primary cellular target of Carolacton is easily saturated and thus of low abundance in cells.

Here we show that growth inhibition by Carolacton occurs for clinical isolates as well as for the virulent laboratory strain TIGR4 and thus appears to be independent of the serotype. Interestingly, the susceptibility to Carolacton was related to the drug resistance of the pathogen. The sepsis isolate Sp49 (which is resistant to two classes of antibiotics only) was instantly killed by Carolacton, while the multidrug-resistant isolates Sp61 and Sp64 were inhibited to a lower degree than the TIGR4 strain. Accordingly, also the MIC values of Carolacton differed notably between *S. pneumoniae* TIGR4 and among the clinical isolates. Carolacton has a potent inhibitory effect on *S. pneumoniae* TIGR4 and Sp49 with MIC values in the range of MICs of classical antibiotics used for the therapy of pneumococcal disease, such as e.g. ampicillin, clarithromycin or rifampicin^31^. The exact MIC of Sp49 remains to be determined and could be even lower than 0.03 μg/ml. Multidrug-resistant isolates Sp61 and Sp64 can tolerate and grow in the presence of high concentrations of Carolacton. Multidrug resistance is often mediated by increased expression or activity of multidrug efflux pumps. Sp61 and Sp64 are resistant to macrolides (clarithromycin, telithromycin)^32^ and fluoroquinolones (levofloxacin)^33^, which is mediated by efflux in the pneumococcus. Carolacton is structurally similar to the macrolide class of natural compounds and might be exported in a similar way. Unspecific efflux of Carolacton over time, as a by-product of resistance to macrolides and fluoroquinolones, could explain why the MICs of Sp61 and Sp64 are much higher than that of *S. pneumoniae* TIGR4. This is in accordance with the observation that *E. coli* is insensitive to Carolacton, but the growth of *E. coli* TolC, which lacks a multidrug efflux pump, is completely inhibited^5^. These findings are important for future development of the compound and potential clinical applications. For example, Carolacton could be used synergistically with structurally unrelated non-macrolide antibiotics.

A Carolacton derivative with an inverted stereocentre at C-9 (*epi*-Carolacton) was completely inactive against both *S. mutans* and *S. pneumoniae*, as well as against several oral streptococci^11^. All other chemical modifications of the side-chain or the lactone ring tested so far were also inactive^11^. This indicates a highly stereospecific interaction of Carolacton with its molecular target and led us to hypothesise that the target could be highly conserved and shared between *S. pneumoniae* and *S. mutans*. It appears unlikely that a switch of the stereochemistry at C-9 could completely abolish interaction with multiple less conserved targets in both species. If Carolacton would interact with several weakly conserved targets, we would expect a remaining activity of *epi*-Carolacton in one of the tested species, which is absent as shown here and previously^11^. A pattern similar to the one described for *epi*-Carolacton here was found for the antibiotic fosfomycin, which targets the conserved bacterial cell wall hydrolase MurA^34^, but its stereoisomer lacks antimicrobial activity^35^. Consequently we investigated the roles of PknB and CysR, which both had been shown to be essential for the damage of biofilm cells of *S. mutans* at low pH^10^,^20^, for Carolacton susceptibility in *S. pneumoniae*. Our data indicate that neither StkP nor CysR are required for susceptibility of *S. pneumoniae* to Carolacton and they thus cannot be its molecular target if this is conserved in both streptococci. Another indication that Carolacton acts independently of StkP is the potent activity of Carolacton against *E. coli* TolC, as *E. coli* does not possess StkP homologues^36^. StkP and CysR are apparently exclusively important for *S. mutans* at low pH in mature biofilms, which is a unique environment fundamentally different from planktonically growing *S. pneumoniae* cultures.

As a possible mode of action of Carolacton, Reck *et al*. proposed a disturbance of cell wall regulatory processes, resulting in plasma membrane damage and leakage of cytoplasmic content at a low pH^10^. Plasma membrane damage is usually paralleled by a loss of membrane potential (MP)^37^. Treatment with antimicrobial agents such as streptomycin, reutericyclin^38^ or ampicillin^37^ can decrease the MP and thereby interfere with the localization of cell division proteins^39^. By fluorescent staining of Carolacton-treated cells with DiOC_2_(3), we detected a continuous decrease in MP over time. When assessing the integrity of the plasma membrane, as a possible cause for MP reduction, no damage could be detected. This is in agreement with Kunze *et al*. who reported no changes in LIVE/DEAD-stained *S. mutans* cells during planktonic growth with Carolacton^8^. Thus, Carolacton treatment leaves the plasma membrane intact in both *S. mutans* and *S. pneumoniae* during planktonic growth.

To identify components of a possible conserved response in *S. mutans* and *S. pneumoniae*, RNA-sequencing was carried out. Among differentially regulated sRNAs, *sspn1731.1* and *sspn1732.1* are strongly downregulated at late time points. Both contain a T-box motif that is known to stimulate anti-termination of the *tyrS* gene in *Bacillus subtilis* by interaction with the acceptor end of tRNA(Tyr)^40^. T-box leader sequences of non-coding RNAs are part of a negative feedback loop controlling expression of aminoacyl-tRNA synthetase genes^41^ and components of the amino acid biosynthesis machinery^42^. Downregulation of *sspn1731.1* and *sspn1732.1* may present another compensatory mechanism to Carolacton by increasing expression, for example of aminoacyl-tRNA-synthetases. Among differentially regulated mRNAs, an immediate strong upregulation of the *sp_0119*-*sp_0120* operon was identified. Both encoded proteins have a global regulatory role in *S. pneumoniae*. The *sp_0119* gene encodes a MutT/Nudix family-like protein. A homologous protein in *E. coli* (MutT) functions as an anti-mutator protein, by maintaining replication fidelity through hydrolysis of mutagenic 8-oxo-dGTP^43^. Other MutT/Nudix family-like proteins in *E. coli* similarly protect the cell by degradation of toxic nucleotide derivatives^44^ and have been described as “housecleaning” enzymes^45^. Thus, it can be assumed that SP_0119 likewise plays a role in protecting the pneumococcus by detoxification of harmful metabolites. GidA (SP_0120) is a highly conserved protein; homologues are present in Proteobacteria and Firmicutes and influence protein biosynthesis by altering mRNA codon recognition through modification of tRNAs at the wobble position^46^. GidA modulates the virulence of a large number of pathogens^47^. Consequently, a *Salmonella gidA* mutant strain is currently being investigated for use in a live-attenuated vaccine^48^. As an important virulence factor, also in streptococci^49^, it is conceivable that by altering the transcription of *gidA*, Carolacton may also interfere with virulence of *S. pneumoniae*. Expression of virulence factors is tightly controlled in group A streptococci^50^ and upregulated transcription of virulence factors can have a negative effect on pathogenicity, as shown for *Listeria monocytogenes*^51^. The investigation of compounds that are aiming at virulence factors in order to render pathogens harmless, instead of targeting essential cellular components, hold enormous possibilities^52^. The use of anti-virulence drugs could even counteract resistance development in bacteria altogether^53^. If *S. pneumoniae* cells show an altered virulence after treatment with Carolacton needs to be explored further.

Growth of single and double deletion mutants of *sp_0119* and *sp_0120* was still impaired by Carolacton, thus neither SP_0119 nor SP_0120 qualify as its molecular target. We hypothesise that these proteins mediate a compensatory response to Carolacton treatment. This is further confirmed by comparison of the transcriptional response of *S. mutans* and *S. pneumoniae*, which suggests that these responses likely show species-specific stress reactions or compensatory mechanisms and indicate that the effect of Carolacton cannot be compensated by differential regulation of a single specific gene set. For example, competence development, e.g. by upregulating transcription of *comDE*, is a direct reaction to stress in bacteria, such as streptococci, that lack a classical SOS response^54^. An upregulation of the VicKRX operon and VicKRX-regulated genes, as seen in *S. mutans*, possibly presents a direct response to disturbance of cell wall homeostasis^55^. The proteins MreC and MreD are located at the equators and septa of dividing *S. pneumoniae* cells and direct peripheral peptidoglycan synthesis^56^. Their increased transcription after Carolacton treatment could be interpreted as an attempt to compensate defects in peptidoglycan synthesis and cell division.

The data show that Carolacton holds promise as a bactericidal antibiotic. The extent of growth inhibition observed after Carolacton treatment in *S. pneumoniae* is variable, does not depend on the serotype and can even be bacteriolytic. This calls for further investigation of the primary mode of action of Carolacton and demonstrates the importance to discover the direct molecular target as a pre-requisite for rational drug design.

## Methods

Additional methods can be found in the Supplementary Information.

### Bacterial strains and growth conditions

Bacterial strains used in this study are listed in Table 2. *S. pneumoniae* TIGR4 serotype 4 (ATCC BAA-334), used for growth experiments in liquid medium, was routinely grown on Columbia blood agar plates containing 5% sheep blood (BD Biosciences) overnight (o/n) at 37 °C and 5% CO_2._ Cells were then scraped from plate and used to inoculate Todd-Hewitt-Broth + 1% (w/v) yeast extract (THBY, BD Biosciences) liquid medium, starting from an optical density at 600 nm (OD_600_) of 0.1. After the initial culture had reached an OD_600_ of 0.15, it was split into equal volumes and Carolacton or 9(*R*) *epi*-Carolacton^11^ were added to a final concentration of 0.25 μg/ml, if not stated otherwise. Carolacton was solved in methanol and stored 1000-fold concentrated (5.3 mM) in small aliquots in glass vials at −20 °C in the dark. *Epi*-Carolacton was solved in dimethyl sulfoxide (DMSO) and also stored in concentrated small aliquots (2 mM) at −20 °C in the dark.

Clinical *S. pneumoniae* isolates and information about clinical background data, serotype and antimicrobial susceptibility were obtained from Dr. Mark van der Linden from the National Reference Laboratory (NRZ) on Streptococcal Disease in Aachen (Germany). All clinical strains were isolated between 5/2008 and 4/2009. For cryo-conservation, *S. pneumoniae* strains were grown in THBY to early exponential phase (OD_600_~0.25) and immediately frozen at −80 °C in 10% (v/v) glycerol.

Growth inhibition was calculated as follows:


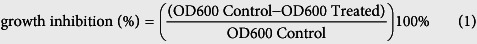


### Determination of MIC values

*S. pneumoniae* was grown on Columbia blood agar plates o/n as described before. Minimum inhibitory concentration (MIC) values of Carolacton were determined by two-fold serial microdilution and incubation at 37 °C and 5% CO_2_ for 18–20 h, as described for the pneumococcus previously^31^. The dilution range of Carolacton tested was 0.03–128 μg/ml. The MIC was determined as the lowest concentration of Carolacton that did not yield visible bacterial growth. The cell number of the initial inoculum was 5 × 10^5^ CFU/ml as determined by serial dilution and plating. Ampicillin, erythromycin, tetracycline and rifampicin were included as controls during MIC determination for *S. pneumoniae* TIGR4, confirming target MIC values commonly reported for susceptible pneumococci^31^. MICs were confirmed by at least two independent measurements (with two replicates).

### Construction of gene deletion mutants of *S. pneumoniae* TIGR4

Construction of gene deletion strains in the TIGR4 background was carried out by the Duplicate, cf. end of sentence. PCR ligation mutagenesis method, replacing the gene of interest (GOI) with an erythromycin (*erm*^*R*^) resistance cassette, via double homologous recombination as described previously^57^. All used enzymes were purchased from New England Biolabs (NEB). First, the up- and downstream regions flanking the GOI were amplified using the primer pairs listed in Supplementary Table S2, introducing an *Fse*I and an *Asc*I restriction site into the PCR products. The *erm*^*R*^ cassette was amplified from pALN122^58^. PCR fragments were digested with the appropriate restriction enzymes and purified using the QIAquick PCR Purification Kit (Qiagen). 100 ng of all DNA fragments was ligated with T4 DNA ligase at 16 °C o/n.

### Transformation of *S. pneumoniae* TIGR4

Transformation of *S. pneumoniae* was carried out by induction of competence by addition of competence stimulating peptide 2 (CSP-2) as described previously^57^. Briefly, THBY (pH 6.8) supplemented with 1 mM CaCl_2_ and 0.2% (w/v) bovine serum albumin (BSA) was inoculated with exponentially growing cells from liquid culture in THBY (OD_600_~0.5) to an OD_600_ of 0.005. After reaching the early log phase (OD_600_~0.15), 1 ml culture was harvested by centrifugation and the cells resuspended in 1 ml THBY (pH 8.0) supplemented with 1 mM CaCl_2_, 0.2% (w/v) BSA and 400 ng/ml CSP-2. Transformation was achieved by addition of 10 μl of a ligation mix and static incubation at 37 °C for 2 h. Transformants were selected on THBY agar plates containing 0.5 μg/ml erythromycin. Correct integration was checked by PCR and Sanger DNA sequencing.

### Verification of the absence of StkP in the TIGR4Δ*stkP* deletion mutant by immunoblotting

In addition to DNA sequencing, the absence of StkP of JD01 was confirmed by immunoblotting. First, a defined volume (V[μl] = 300/OD_600_) of *S. pneumoniae* TIGR4 wild type and TIGR4Δ*stkP* cells in THBY was boiled in SDS loading dye (95 °C, 15 min) and separated via 12% (v/v) SDS PAGE according to^59^. Proteins were blotted to a PVDF membrane (Immobilion-P, Milipore) using a semi-dry blotting system (Sigma-Aldrich) and blocked with 5% (w/v) BSA in TBS-T at room temperature (RT) for 1 h. Incubation with the primary antibody, rabbit anti-StkP, was carried out by using a 1:50.000 dilution in 1% (w/v) BSA/TBS-T o/n. Pneumococcal α-enolase (α-eno) served as a loading control^60^. The α–eno antibody was used in a 1:500 dilution in 1% (w/v) BSA/TBS-T at RT o/n. Alkaline phosphatase-coupled goat anti-rabbit IgG (Sigma-Aldrich) diluted 1:10 000 in TBS-T was applied for 1 h at RT for detection of the primary antibody. Alkaline phosphatase activity was visualized colorimetrically using SIGMA FAST BCIP/NBT tablets according to the manufacturer’s instructions (Sigma-Aldrich).

### Isolation and enrichment of mRNA

Processing of RNA-seq data is presented in detail in the Supplementary Information. Total RNA from was isolated using the miRNeasy mini kit (Qiagen) according to the manufacturer’s instructions for separation of small RNA-enriched fractions (≤200 nt) and larger transcripts (>200 nt). DNA library generation was carried out using the ScriptSeq™ v2 RNA-Seq Library Preparation Kit (epicentre) for long transcripts (>200 nt), and the TruSeq Small RNA Library Prep Kit (Illumina) for transcripts ≤200 nt. Libraries were sequenced on the Illumina HiSeq 2500 (Illumina) using 50-bp single-end sequencing.

### RNA-seq data analysis

Processing of RNA-seq data is described in Supplementary Information. All analysed small RNAs are documented in Supplementary Dataset S3. False discovery rate (FDR)-adjusted P values were calculated according to^61^. FDR values of <0.01 were considered significant. For visualization of these results heat maps were generated, taking into account only genes that, in addition to a significant FDR, showed a log_2_-fold change (log_2_FC) of transcription of ≥ ± 2 (or ≥ ± 0.8 for small RNAs) at least at one point during a time course. For this, the log_2_-fold values of transcript abundance obtained in edgeR were used in combination with the heatmap. 2 function of the R package gplots (v2.15.0)^62^. Raw and processed RNA-seq data have been deposited in NCBI’s Gene Expression Omnibus (GEO) database^63^ and are accessible through GEO Series accession number GSE76979.

### Fluorescent staining of *S. pneumoniae*

To detect changes in the membrane potential of *S. pneumoniae* TIGR4 when treated with Carolacton, the bacteria were stained with the fluorescent dye *3*,*3*-Diethyloxacarbocyanine iodide (DiOC_2_(3)) (*Bac*Light Bacterial Membrane Potential Kit, Life Technologies). For staining with DiOC_2_(3), cells were diluted from culture to approx. 10^7^ cells/ml and stained with 30 μM DiOC_2_(3) in the dark for 30 min. In order to assess bacterial membrane integrity, cells were separately co-stained with the fluorescent dyes Syto 9 and propidium iodide (PI) using the LIVE/DEAD *Bac*Light Bacterial Viability Kit (Life Technologies).

For Syto 9/PI staining, cells from a culture grown with 0.25 μg/ml Carolacton and from a control were harvested by centrifugation and washed in 0.85% (w/v) NaCl. Approx. 5 × 10^6^ cells/ml were stained with an equal volume 2× staining solution (10 μM Syto 9, 60 μM PI) at RT in the dark for 30 min. Heat-inactivated cells were used as a control in both staining experiments. Heat-killing of cells was achieved by incubation at 80 °C and shaking (1,400 rpm) for 30 min.

### Flow cytometric analysis of fluorescently-labelled *S. pneumoniae* cells

DiOC_2_(3) and Syto 9/PI-fluorescently stained cells were analysed in triplicate on a FACSCanto flow cytometer (BD Biosciences) as described in the material section of the Supplementary Information.

### Microscopy and quantification of cell size

*S. pneumoniae* TIGR4 cells growing in THBY were washed with 0.85% (w/v) NaCl solution and mounted to a microscope slide coated with a thin layer of 1% (w/v) agarose. Phase-contrast microscopic analysis of *S. pneumoniae* was performed using an Olympus BX60 microscope equipped with an Olympus DP50 color camera (5 Mpixel) color view II camera and a 100x magnification oil immersion objective. Pictures and measurements were taken with the Olympus cellSens (v1.6) microscopy software. The length of pneumococcal cells was measured along the length axis. Cell width was determined as the diameter along the equatorial plane, perpendicular to the length axis of the cell.

### Quantification of colony forming units

For determination of colony forming units (CFUs), cells were treated with Carolacton (0.25 μg/ml) prior to exponential growth. Ten-fold serial dilutions of cultures were prepared in 0.85% (w/v) NaCl solution (pre-warmed to 37 °C) after 3 h and 6 h of growth. A total volume of 100 μl from each dilution was then plated onto pre-warmed (37 °C) THBY agar plates, which were incubated at 37 °C and 5% CO_2_ o/n.

### Statistical analysis

Comparisons between two groups at a single time were performed with unpaired two-tailed (non-parametric) Mann-Whitney U test^64^ or with Student’s t-tests for two-tailed distribution (with a critical *P* value of 0.05). The data were analysed for normality by Shapiro-Wilk test^65^. Statistical analyses were performed with the statistics options of SigmaPlot (v13.0) software (Systat Software Inc.).

## Additional Information

Accession codes: Raw and processed RNA-seq data have been deposited in NCBI’s Gene Expression Omnibus. (GEO) database and are accessible through GEO Series accession number GSE76979. 

**How to cite this article**: Donner, J. *et al*. The biofilm inhibitor Carolacton inhibits planktonic growth of virulent pneumococci via a conserved target. *Sci. Rep.* **6**, 29677; doi: 10.1038/srep29677 (2016).

## Supplementary Material

Supplementary Information

Supplementary Dataset 1

Supplementary Dataset 2

Supplementary Dataset 3

## Figures and Tables

**Table 1:** Minimal inhibitory concentration (MIC) of Carolacton against planktonically growing *S. pneumoniae* strains.

Strain	Carolacton (μg/ml)
TIGR4	0.06
Sp49 (NRZ:3198/36486)	≤ 0.03
Sp61 (NRZ:3364/39533)	64
Sp64 (NRZ:3066/35051)	64

**Table 2:** Bacterial strains, clinical isolates and plasmids used in this study.

Strain/plasmid	Relevant genotype/serotype	Description	Reference
*Streptococcus pneumoniae*			
TIGR4 (ATCC BAA-334)	encapsulated serotype 4	wild type	19
JD01	TIGR4 *sp_1732*::*erm*^*R*^	Δ*stkP* strain	This work
JD02	TIGR4 *sp_0927*::*erm*^*R*^	Δ*cysR* strain	This work
JD03	TIGR4 *sp_0119*::*erm*^*R*^	Δ*sp_0119* strain	This work
JD04	TIGR4 *sp_0120*::*erm*^*R*^	Δ*gidA* strain	This work
JD05	TIGR4 *sp_0119-sp_0120*::*erm*^*R*^	Δ*sp_0119/*Δ*gidA* strain	This work
*Clinical isolates*			
Sp49	serotype 19A	pneumonia/sepsis isolate	M. van der Linden, NRZ Aachen
Sp61	serotype 19A	pleuritis isolate	M. van der Linden, NRZ Aachen
Sp64	serotype 19A	mid-ear isolate	M. van der Linden, NRZ Aachen
*Plasmid*			
pALN122	*erm*^*R*^		58

**Table 3:** Summary of the most differentially expressed genes upon growth of *S. pneumoniae* TIGR4 with Carolacton for 180 min.

Locus tag	Gene product	Gene symbol	log_2_FC^a^	FDR
*Galactose uptake* (*PTS*)*/N-acetylgalactosamine uptake* (*PTS*)*/Protein export*				
* sp_0324*	PTS system transporter subunit IIC		4.68	7.52E-91
* sp_0325*	PTS system transporter subunit IID		4.56	5.10E-123
* sp_0322*	glucuronyl hydrolase		4.73	2.58E-100
* sp_0327*	hypothetical protein		4.41	5.05E-74
* sp_0323*	PTS system transporter subunit IIB		4.80	2.75E-95
* sp_0326*	preprotein translocase subunit	*yajC*	4.55	5.11E-74
* sp_0321*	PTS system transporter subunit IIA		4.96	6.23E-89
*Carbon metabolism/Pentose phosphate pathway*				
* sp_0318*	carbohydrate kinase		4.13	1.30E-67
* sp_0320*	gluconate 5-dehydrogenase		4.08	1.82E-78
* sp_0317*	4-hydroxy-2-oxoglutarate aldolase/2-dehydro-3-deoxyphosphogluconate aldolase		3.82	6.60E-64
* sp_0319*	hypothetical protein		3.88	4.69E-72
*Protein degradation*				
* sp_0338*	ATP-dependent Clp protease, ATP-binding subunit		3.73	4.09E-67
*MutT/nudix family protein/transfer RNA biogenesis*				
* sp_0119*	MutT/nudix family protein		4.44	2.57E-67
* sp_0120*	tRNA uridine 5-carboxymethylaminomethyl modification protein	*gidA*	4.33	1.35E-50
*Phosphate* (*P*_*i*_) *uptake* (*ABC transporter*)				
* sp_2087*	phosphate transporter ATP-binding protein	*pstB*	2.96	1.16E-45
* sp_2086*	phosphate ABC transporter permease	*pstA*	2.97	1.34E-42
* sp_2084*	phosphate ABC transporter permease	*pstS*	2.89	2.78E-38
* sp_2085*	phosphate ABC transporter substrate-binding protein	*pstC*	2.96	2.78E-33
* sp_2088*	phosphate transport system regulatory protein	*phoU*	3.19	5.26E-42
* sp_0789*	conserved hypothetical protein		2.64	2.13E-37
* sp_0790*	conserved hypothetical protein		2.58	2.77E-44
* sp_0018*	hypothetical protein		2.10	1.13E-24
*Genetic competence*				
* sp_2207*	competence protein	*comFC*	2.21	2.18E-05
* sp_2208*	helicase	*comFA*	2.22	3.04E-09
* sp_1793*	hypothetical protein		2.17	9.19E-32
* sp_1777*	hypothetical protein		2.22	2.46E-25
* sp_2013*	hypothetical protein		2.13	4.64E-31
*Nicotinate and nicotinamide metabolism*				
* sp_2015*	IS630-Spn1, transposase Orf1		2.74	2.48E-40
* sp_2016*	nicotinate-nucleotide pyrophosphorylase		2.60	1.07E-46
* sp_0539*	bacteriocin	*blpM*	2.59	1.38E-04
*Starch and sucrose metabolism*				
* sp_1722*	PTS system IIABC components		0.87	6.04E-04
* sp_1314*	IS66 family element, Orf1		−3.11	7.89E-03
* sp_1803*	hypothetical protein		−1.35	3.02E-09
*Central carbon/lipid metabolism*				
* sp_0252*	fructose-6-phosphate aldolase		−2.34	4.59E-06
* sp_0253*	glycerol dehydrogenase		−2.57	2.21E-07
* sp_0251*	formate acetyltransferase		−2.29	8.60E-06
*Cellobiose uptake* (*PTS*)				
* sp_0248*	PTS system transporter subunit IIA		−2.30	8.11E-05
* sp_0250*	PTS system transporter subunit IIC		−2.28	1.85E-05
* sp_0249*	PTS system transporter subunit IIB		−2.42	4.79E-05

Horizontal lines indicate separation of transcriptional units.

^a^cut-off: log_2_FC≥2 or ≤−2 at one point during the time course.
