# Supplementary material for: The biofilm inhibitor Carolacton inhibits planktonic growth of virulent pneumococci via a conserved target
Source: Sci Rep. 2016 Jul 11;6:29677. doi: 10.1038/srep29677 (PMC4939601; doi:10.1038/srep29677)
Supplement: Supplementary Information [file srep29677-s1.pdf]

Supplementary Information

The biofilm inhibitor Carolacton inhibits planktonic growth of virulent pneumococci via a conserved target

Jannik Donner[1]*, Michael Reck[1], Simone Bergmann[2], Andreas Kirschning[3], Rolf Müller[4], and Irene Wagner-Döbler[1]

[1]Department of Medical Microbiology, Group Microbial Communication, Helmholtz Centre for Infection Research, Braunschweig, Germany

[2]Institute of Microbiology, Technische Universität Braunschweig, Braunschweig, Germany

[3]Institute of Organic Chemistry and Center of Biomolecular Drug Research (BMWZ), Leibniz Universität Hannover, Hannover, Germany

[4]Department of Microbial Natural Products, Helmholtz Institute for Pharmaceutical Research Saarland (HIPS), Helmholtz Centre for Infection Research and Pharmaceutical Biotechnology, Saarland University, Saarbrücken, Germany.

*Corresponding author: Jannik Donner, jannik.donner@helmholtz-hzi.de

Supplementary Figures and Tables

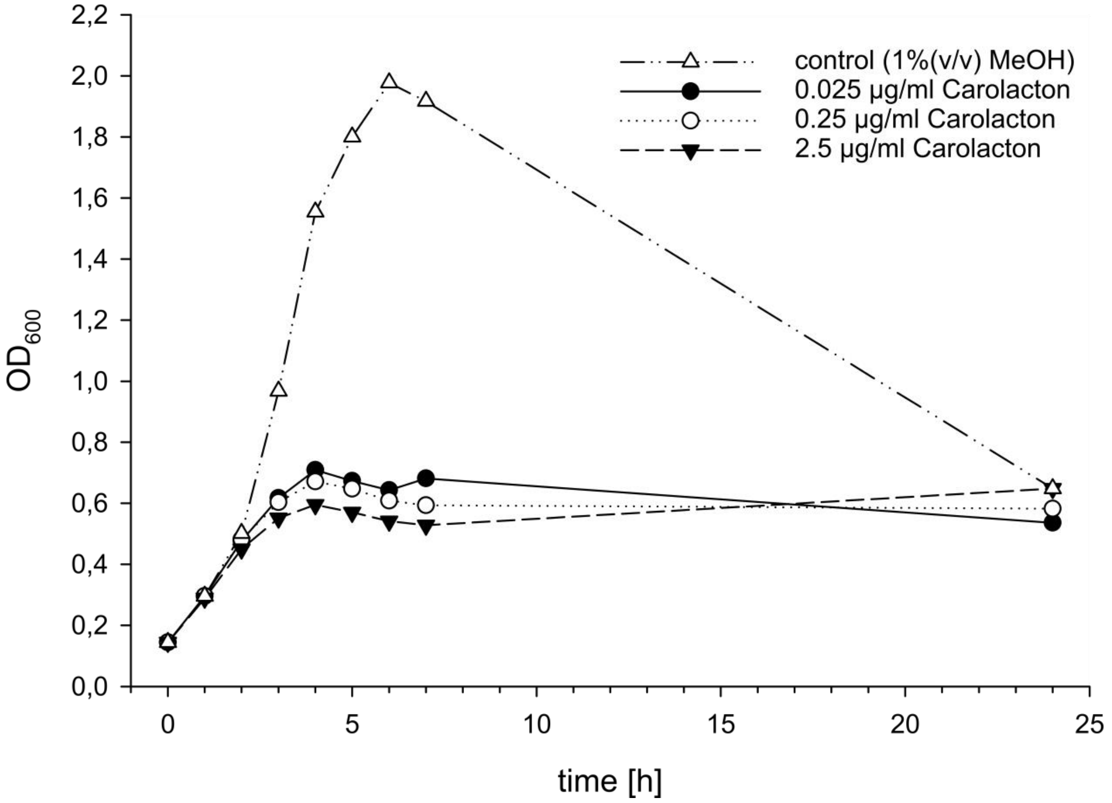

Supplementary Figure S1. Concentration-independent growth-inhibition of *S. pneumoniae* TIGR4 by Carolacton. Shown are the growth-response curves for cells that were grown with 2.5, 0.25 and 0.025 µg/ml Carolacton, corresponding to 53, 5.3 and 0.53 µM, respectively. The shown Supplementary Figure is representative for three independent biological replicates.

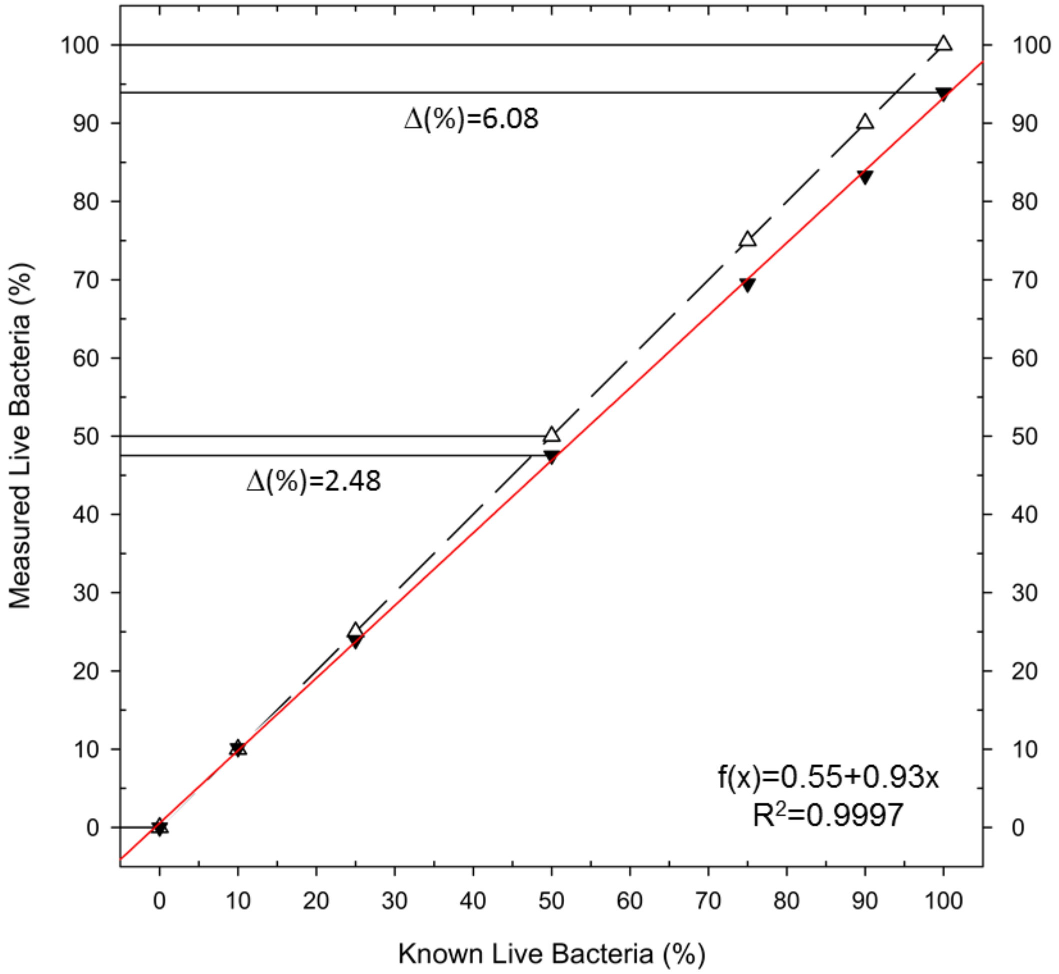

Supplementary Figure S2. Assessment of applicability of Syto 9/PI staining for quantification of membrane damage of *S. pneumoniae* TIGR4 by flow cytometry. Heat-killed *S. pneumoniae* TIGR4 cells, were mixed with live cells obtained from exponentially growing culture in different ratios, stained with Syto 9/PI and analysed via flow cytometry (black triangles). A linear regression analysis was used to draw a trend line (red line, f(x)), R^2=0.9997), clearly indicating a close linear relationship between the data points. The calculated least squares fit was further extrapolated to the point containing 100% live bacteria (white triangles), suggesting a maximum of 6.08% dead cells in the 100% live population.

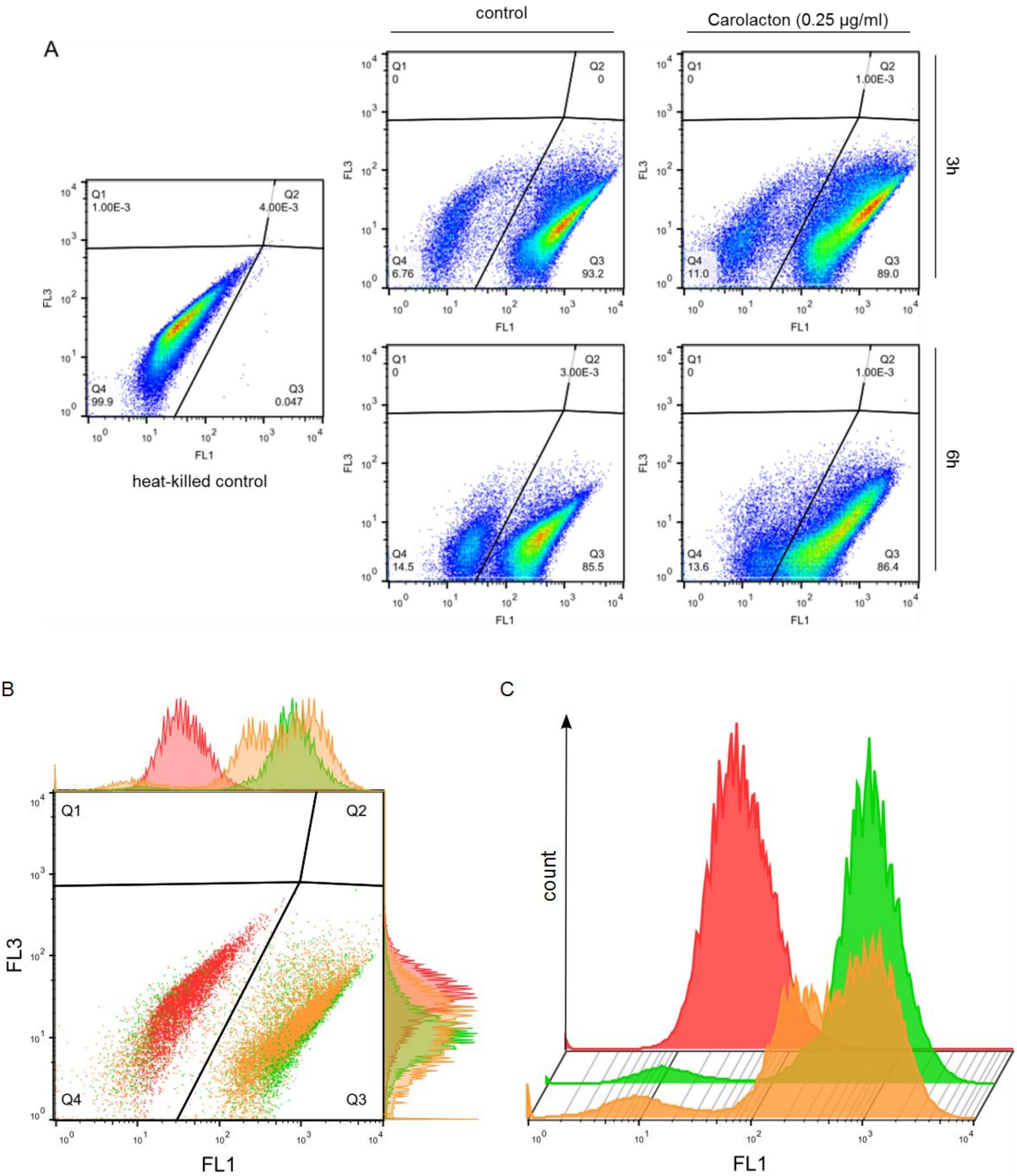

Supplementary Figure S3. Flow cytometric analysis of Syto 9/PI-stained cells grown with Carolacton. (A) Shown are dot plots of Syto 9/PI-stained *S. pneumoniae* TIGR4 cells after 3h

and 6h of growth with 0.25 µg/ml Carolacton and a control. Syto 9/PI-stained heat-killed cells were used to identify the dead population (bottom left). The percentages of cells identified in the different quadrants of the plots (Q1-4) are shown in the respective corners. **(B)** Example of the shift in recorded fluorescence intensities (FL1/FL3) of Carolacton (0.25 µg/ml)-treated and Syto 9/PI-stained cells after 3h. Colouring: green (untreated control); orange (Carolacton-treated); red (heat-killed cells). **(C)** Exemplary histogram of recorded green fluorescence (FL1) intensities versus relative cell count of *S. pneumoniae* TIGR4 cells after 3h. Colouring as shown in (B). The figure stands representative for three independent experiments.

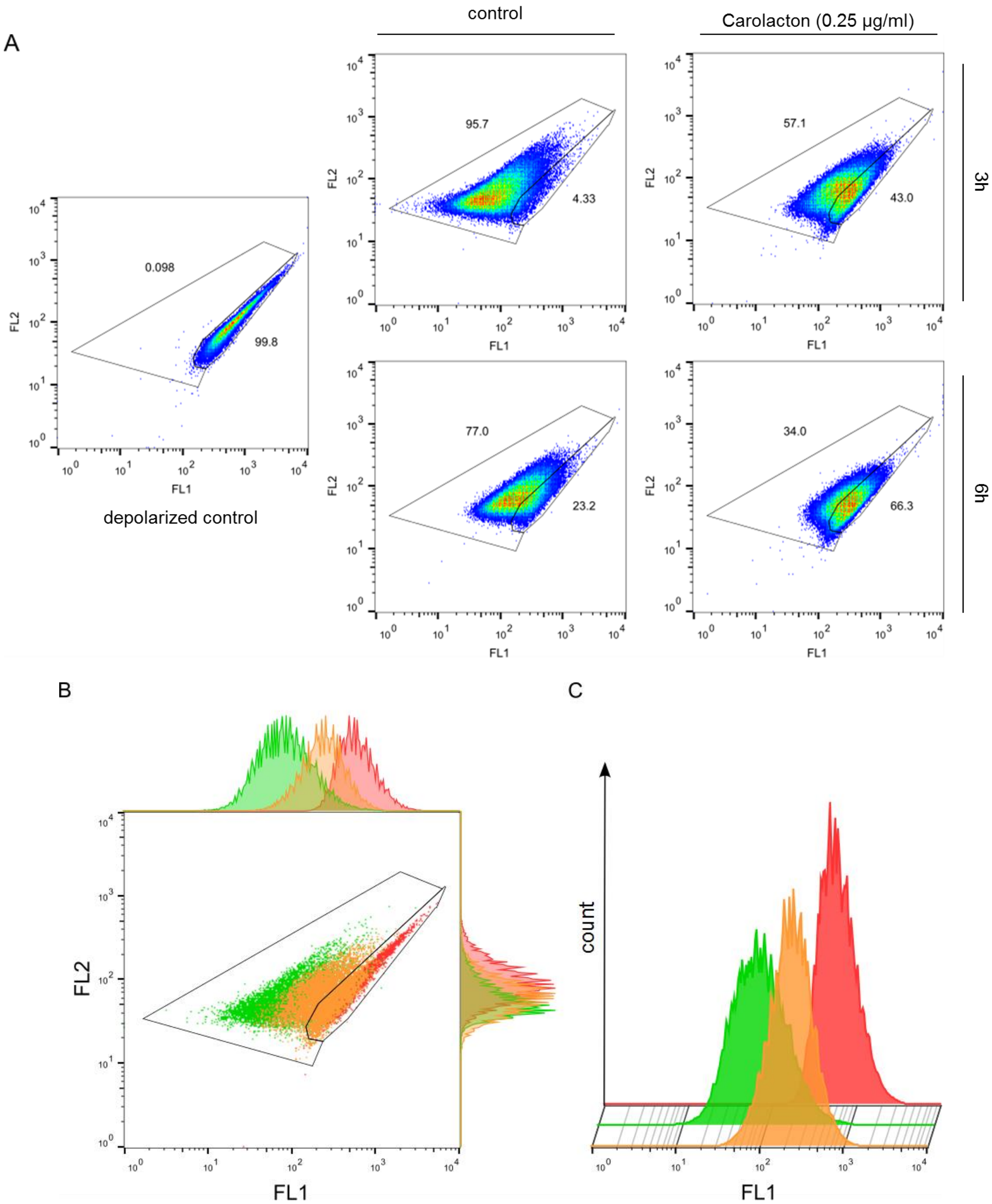

Supplementary Figure S4. Flow cytometric analysis of DiOC$_2$(3)-stained cells grown with Carolacton. (A) Shown are dot plots of DiOC$_2$(3)-stained *S. pneumoniae* TIGR4 cells after 3h

and 6h of growth with 0.25 µg/ml Carolacton and a control. $DiOC_2(3)$-stained heat-killed cells were used to identify the depolarized population (left). The percentages of cells identified in the different quadrants of the plots (Q1-4) are shown in the respective corners. **(B)** Example of the shift in recorded fluorescence intensities (FL1/FL2) of Carolacton (0.25 µg/ml)-treated and $DiOC_2(3)$-stained cells after 3h. Colouring: green (untreated control); orange (Carolacton-treated); red (heat-killed cells). **(C)** Histogram showing the recorded green fluorescence (FL1) intensities versus relative cell counts of *S. pneumoniae* TIGR4 cells after 3h. Colouring as shown in (B). The figure stands representative for three independent experiments.

A
tRNAs

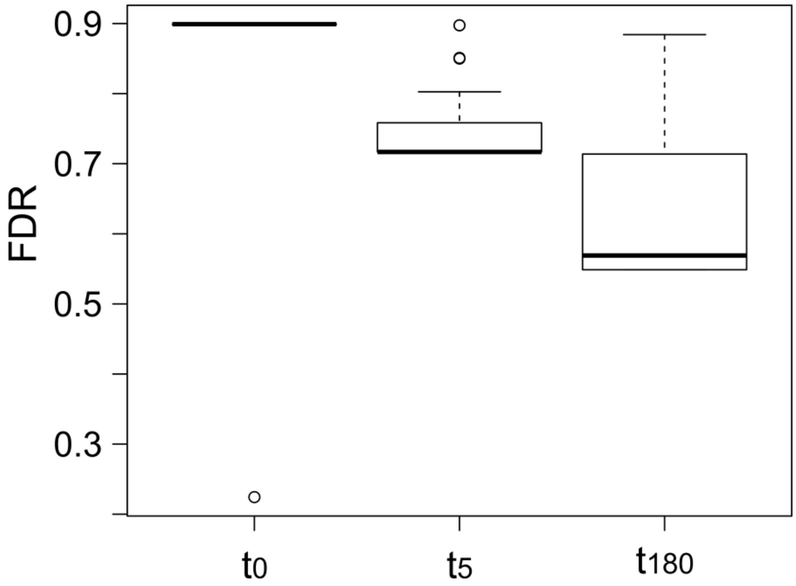

B
sRNAs ≤200 nt

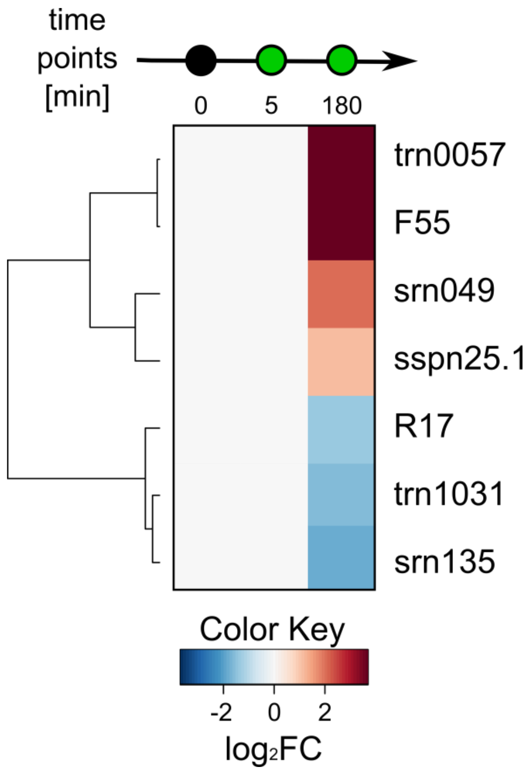

C
sRNAs >200 nt

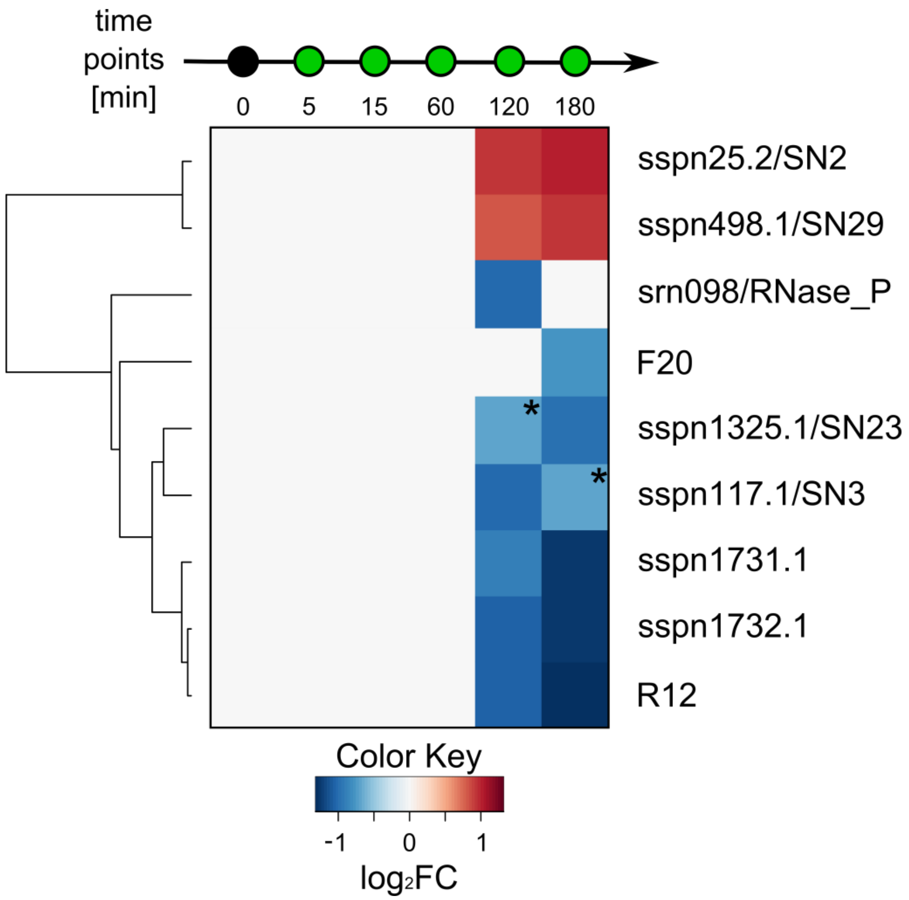

D
mRNAs ≤200nt

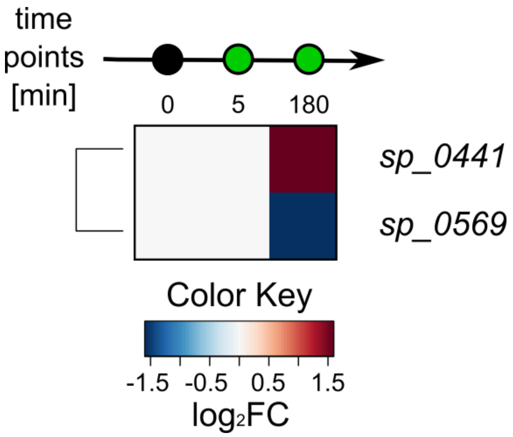

Supplementary Figure S5. Analysis of different cellular RNA species of *S. pneumoniae* TIGR4 for differential expression after treatment with 0.25 µg/ml Carolacton. (A) Analysis of differential expression of tRNAs, $log_2FC \geq \pm 0.8$, FDR<0.01. **(B)** Heat map of differentially

regulated sRNAs ≤200 nt; $\log_2$FC≥±0.8, FDR<0.01; “*”=significant differentially regulated transcripts with FDR<0.01 but $\log_2$FC<±0.8, $\log_2$FC($SN23_{t120}$)=-0.737; $\log_2$FC($SN3_{t180}$)=-0,735. **(C)** Differentially expressed small sRNAs (>200 nt), $\log_2$FC≥±0.8, FDR<0.01. **(D)** Differentially expressed small mRNAs (≤200 nt), $\log_2$FC≥±0.8, FDR<0.01. White fields within heat maps indicate lack of significance (FDR>0.01) at this time point. Differential expression was calculated using the Bioconductor package edgeR for R.

A **B**

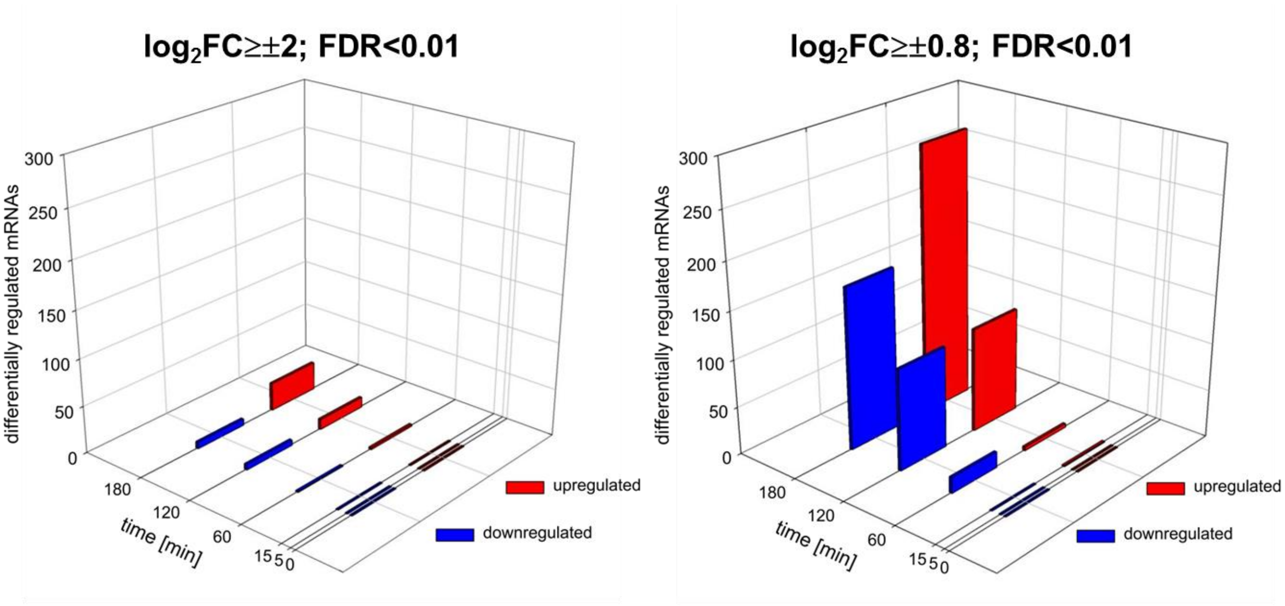

Supplementary Figure S6. Total number of differentially regulated mRNAs after treatment of *S. pneumoniae* TIGR4 with Carolacton. Comparison of the total number of all differentially regulated mRNAs during growth with 0.25 µg/ml Carolacton at the indicated time points for different cut-offs of the log_2FC change: ≥±2 **(A)** and ≥±0.8 **(B)**.

A

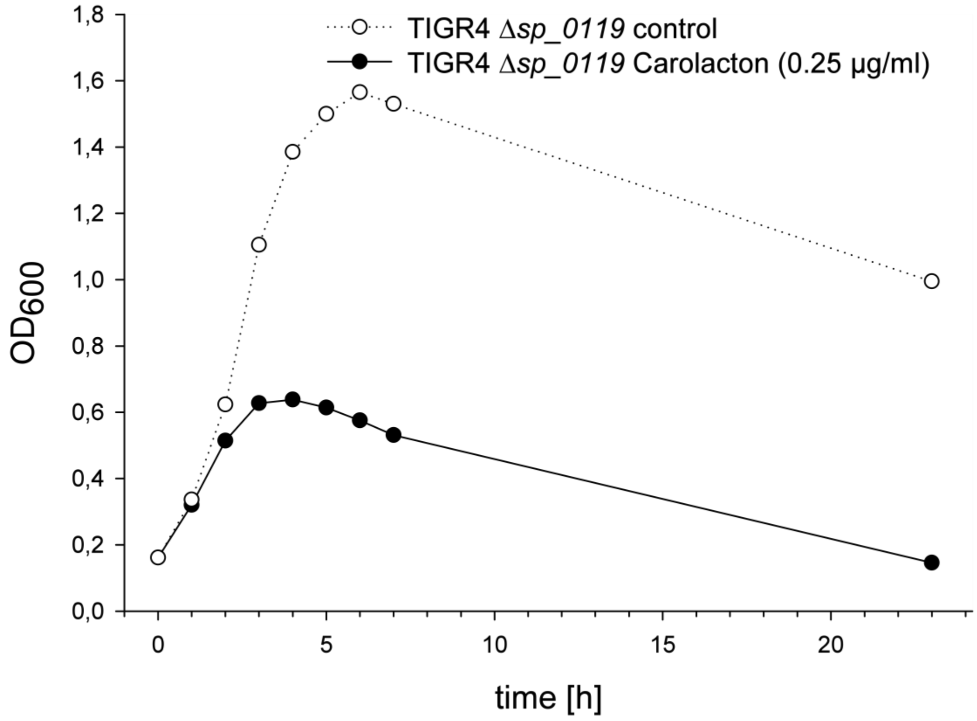
TIGR4 Δsp_0119 control
TIGR4 Δsp_0119 Carolacton (0.25 µg/ml)
OD600
time [h]
1,8
1,6
1,4
1,2
1,0
0,8
0,6
0,4
0,2
0,0
0
5
10
15
20

B

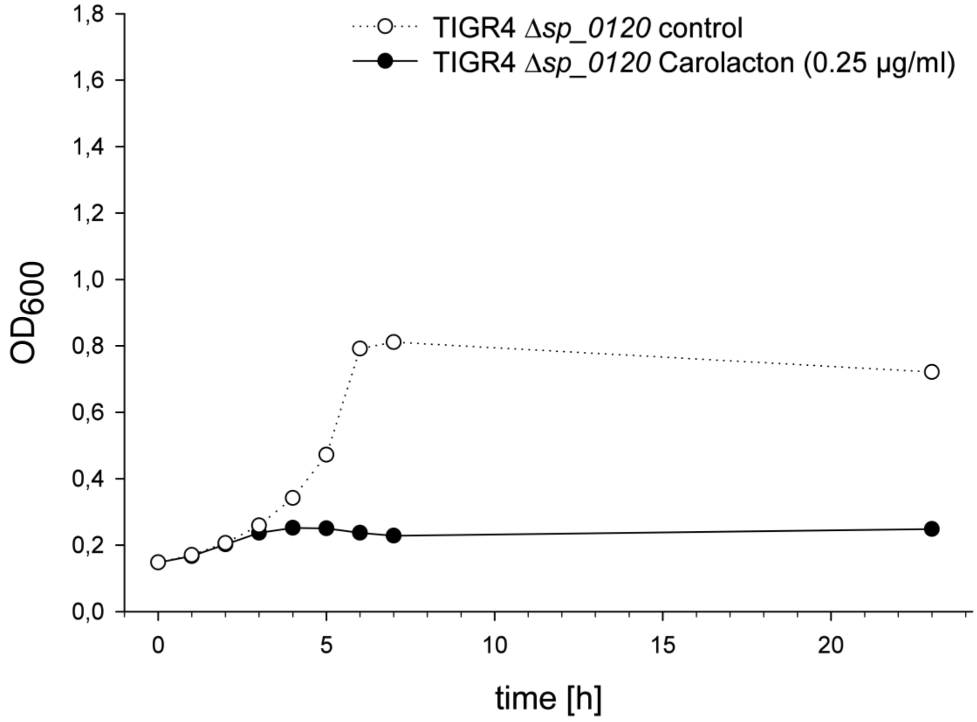
TIGR4 Δsp_0120 control
TIGR4 Δsp_0120 Carolacton (0.25 µg/ml)
OD600
time [h]
1,8
1,6
1,4
1,2
1,0
0,8
0,6
0,4
0,2
0,0
0
5
10
15
20

C

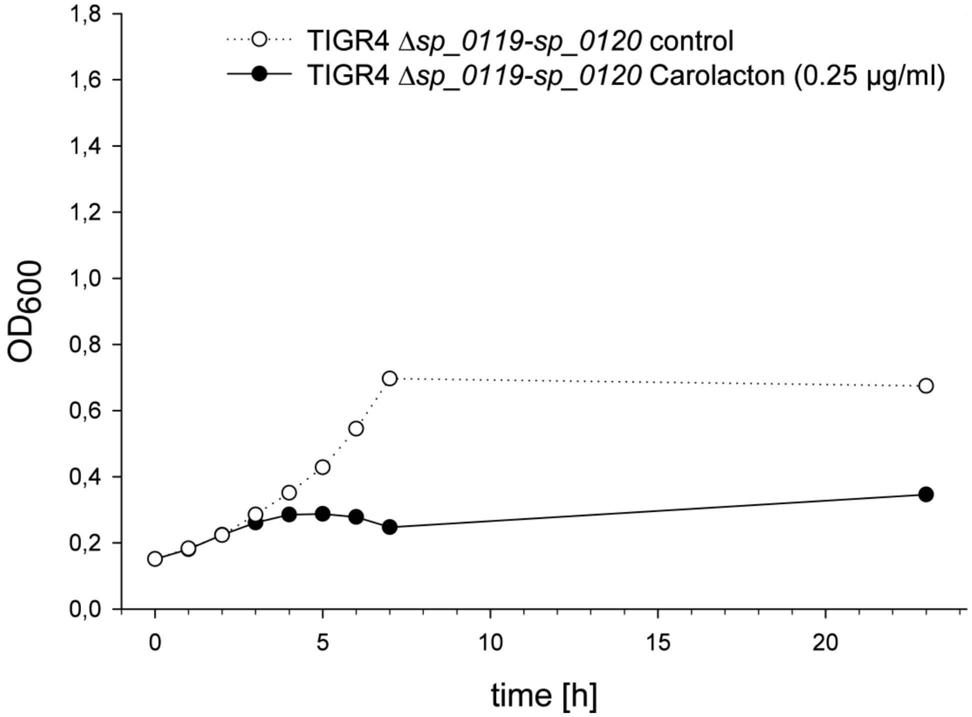

Supplementary Figure S7. Growth response of *S. pneumoniae* TIGR4 *sp_0119*/*sp_0120* single deletion mutants and a *sp_0119-sp_0120* double deletion mutant to Carolacton. Mutants were grown in THBY in the presence of 0.25 µg/ml Carolacton. Single deletion mutants of *sp_0119* **(A)** and *sp_0120* **(B)**, as well as the *sp_0119-sp_0120* double mutant **(C)**, were inhibited by Carolacton. Deletion of *sp_0120* resulted in a notable growth defect (B, C) when compared to the TIGR4 wild type (Supplementary Figure S1) or the *sp_0119* k.o. mutant (A). The shown growth curves stand representative for three biological replicates.

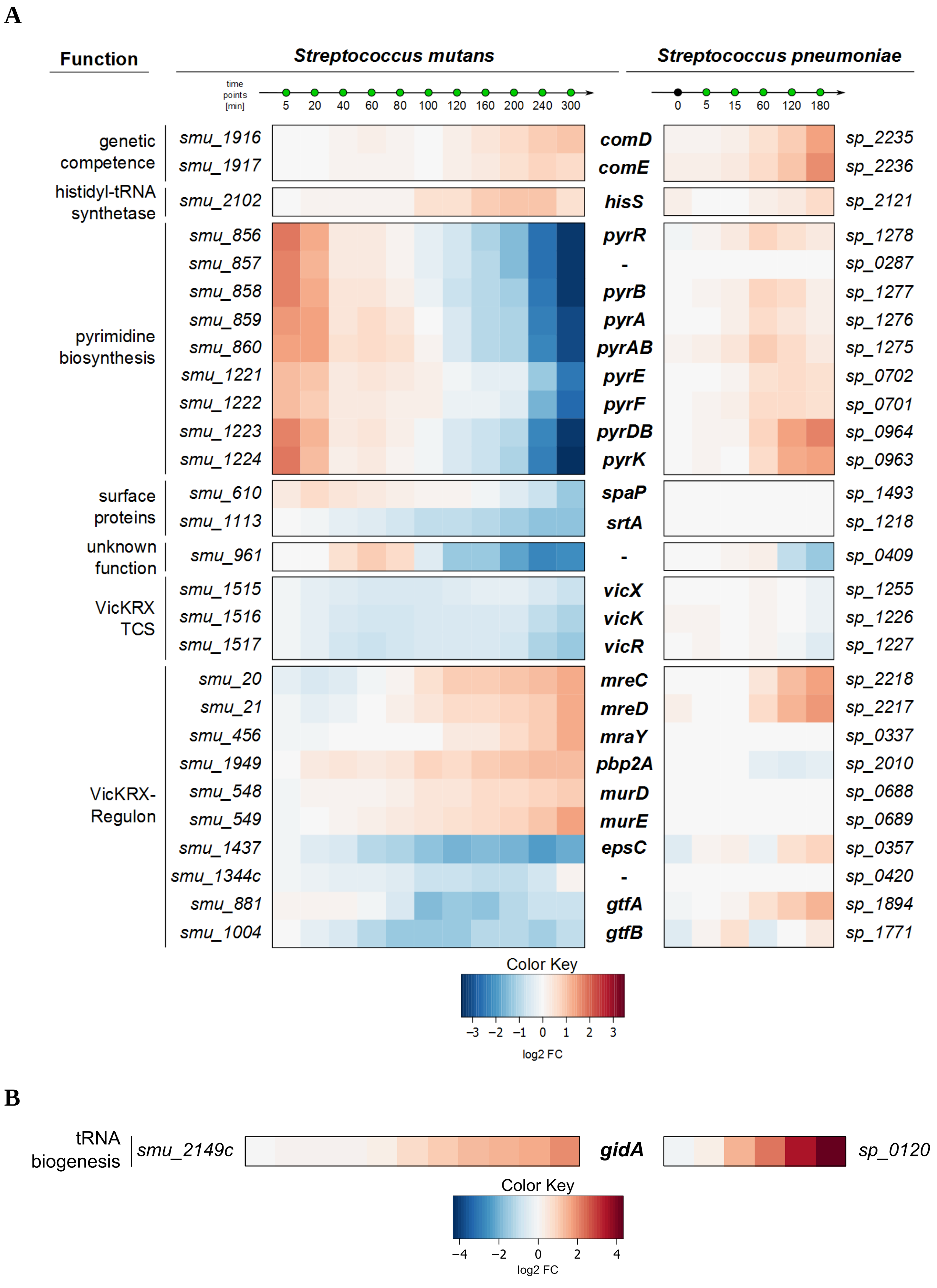
A
Function
Streptococcus mutans
Streptococcus pneumoniae
time points [min]
5 20 40 60 80 100 120 160 200 240 300
0 5 15 60 120 180
genetic competence
smu_1916 comD sp_2235
smu_1917 comE sp_2236
histidyl-tRNA synthetase
smu_2102 hisS sp_2121
pyrimidine biosynthesis
smu_856 pyrR sp_1278
smu_857 - sp_0287
smu_858 pyrB sp_1277
smu_859 pyrA sp_1276
smu_860 pyrAB sp_1275
smu_1221 pyrE sp_0702
smu_1222 pyrF sp_0701
smu_1223 pyrDB sp_0964
smu_1224 pyrK sp_0963
surface proteins
smu_610 spaP sp_1493
smu_1113 srtA sp_1218
unknown function
smu_961 - sp_0409
VicKRX TCS
smu_1515 vicX sp_1255
smu_1516 vicK sp_1226
smu_1517 vicR sp_1227
VicKRX-Regulon
smu_20 mreC sp_2218
smu_21 mreD sp_2217
smu_456 mraY sp_0337
smu_1949 pbp2A sp_2010
smu_548 murD sp_0688
smu_549 murE sp_0689
smu_1437 epsC sp_0357
smu_1344c - sp_0420
smu_881 gtfA sp_1894
smu_1004 gtfB sp_1771
Color Key
−3 −2 −1 0 1 2 3
log2 FC
B
tRNA biogenesis
smu_2149c gidA sp_0120
Color Key
−4 −2 0 2 4
log2 FC

Supplementary Figure S8. Heat map-comparison of differentially transcribed genes between *S. mutans* UA159 and *S. pneumoniae* TIGR4 upon treatment with Carolacton. Data for differential gene expression of *S. mutans* was obtained from time-series microarray experiments conducted by Reck *et al*.[1]. *S. mutans*, as well as *S. pneumoniae* were treated with a final concentration of 0.25 µg/ml Carolacton. **(A)** Comparison of significant differentially regulated genes, operons and regulatory components in *S. mutans* grown with Carolacton (as described by Reck *et al*., 2011) to Carolacton-treated *S. pneumoniae*. **(B)** Differential transcription of the *gidA*-transcript in *S. mutans*. GidA is the earliest and one of the most strongly upregulated transcripts in *S. pneumoniae* when grown with Carolacton. It is involved in biogenesis (formation of wobble 5-carboxymethylaminomethyluridine) of tRNAs.

Supplementary Table S1. Characteristics and susceptibility of the tested clinical *S. pneumoniae* isolates of serotype 19A to antibiotics.

Isolate	Sero-type	Diagnosis	Source	Antimicrobial Agent	MIC (µg/ml)	Interpretation [a,b]
Sp49 (NRZ:3198/36486)	19A	pneumonia/ sepsis	blood	Oxacillin[#]	-	resistant
				Penicillin	0.5	intermediate
				Cefotaxime	0.12	susceptible
				Clarithromycin	0.12	susceptible
				Clindamycin	0.12	susceptible
				Tetracycline	0.5	susceptible
				Telithromycin	0.03	susceptible
				Levofloxacin	1	susceptible
				Moxifloxacin	0.12	susceptible
				Chloramphenicol	4	susceptible
				Trimethoprim/ Sulfamethoxazole*	4	resistant
				Vancomycin	0.5	susceptible
Sp61 (NRZ:3364/39533)	19A	pleuritis	thorax drainage	Oxacillin[#]	-	resistant
				Penicillin	4	resistant
				Cefotaxime	1	intermediate
				Clarithromycin	16	resistant
				Clindamycin	16	resistant
				Tetracycline	16	resistant
				Telithromycin	0.25	intermediate
				Levofloxacin	1	susceptible
				Moxifloxacin	0.25	susceptible
				Chloramphenicol	4	susceptible
				Trimethoprim/ Sulfamethoxazole*	8	resistant
				Vancomycin	0.5	susceptible
Sp64 (NRZ:3066/35051)	19A	-	swab	Oxacillin[#]	-	resistant
				Penicillin	4	resistant
				Cefotaxime	2	resistant
				Clarithromycin	16	resistant

Clindamycin	16	resistant
Tetracycline	16	resistant
Telithromycin	0.06	susceptible
Levofloxacin	1	susceptible
Moxifloxacin	0.25	susceptible
Chloramphenicol	4	susceptible
Trimethoprim/ Sulfamethoxazole*	8	resistant
Vancomycin	0.5	susceptible

[a] Clinical and Laboratory Standards Institute, Performance Standards for Antimicrobial Susceptibility Testing;
Twenty-Fourth Informational Supplement. CLSI document M100-S24, 2014.
[b] British Society for Antimicrobial Chemotherapy, Methods for Antimicrobial Susceptibility Testing, v.12, 2013.
[#] Susceptibility to oxacillin tested by oxacillin (1 µg) disc assay. Resistant = inhibition zone diameter ≤10 mm.
* The MIC is based on the concentration of trimethoprim (in a combination of 1:19 with sulfamethoxazole).

Supplementary Table S2. Overview of used oligonucleotides.

**Primer**	**Sequence (5'→3')[a]**	**Use**	**Restriction sites; Reference**
stkp_up_fwd	CAGTCAATTTCGCAAGATATCGG ATTAGGAAGGAACTGACACATGG	Δ*sp_1732*	This work
stkp_up_rev	AAA*GGCCGGCC*TAGACATCCGCC ATACCTCCTCGACCAATCTGT	Δ*sp_1732*	*Fse*I; this work
stkp_down_fwd	AAA*GGCGCGCC*GTAGTGCAGAAG GCATGGTTGTTGAACAAAGTCCT AGAGC	Δ*sp_1732*	*Asc*I; this work
stkp_down_rev	CGGCATGACCGCACCAGCTATAA TCAAATCTGC	Δ*sp_1732*	This work
cysR_up_fwd	CGGAGTTCTTTTCTTAGCGGTCAT CTATCTACTGGTTTTTGCAGG	Δ*sp_0927*	This work
cysR_up_rev	AAA*GGCCGGCC*GAACAGAAATAG ACAGACTCGGCTGACTAACATAC ATCTTTTCAGCAGC	Δ*sp_0927*	*Fse*I; this work
cysR_down_fwd	AAA*GGCGCGCC*GCATGGTCTATG TTAAACGTGAAGAAGTGGAGCTT	Δ*sp_0927*	*Asc*I; this work

	AGTCAAGC		
cysR_down_rev	CCAGCACTCGGGTGCACAACCATTCC	Δ*sp_0927*	This work
sp_0119_up_fwd	TAAGGTGACCGTTCATGTCAAAGGAGAAAAGACAGAGG	Δ*sp_0119*/Δ*sp_0119-sp_0120*	This work
sp_0119_up_rev	AAA*GGCCGGCC*GGTAACTAGAAGCTTGTGATTTTGGACAATCAAGGCTGTAGC	Δ*sp_0119*/Δ*sp_0119-sp_0120*	*Fse*I; this work
sp_0119_down_fwd	AAA*GGCGCGCC*AGCTAGTTCCAGTCTTTTTAAAAACAGCCCTACCAGATTGG	Δ*sp_0119*	*Asc*I; this work
sp_0119_down_rev	CGGTGGAGGTTGTTTTGGATAATCTCATGACTGGTACC	Δ*sp_0119*	This work
sp_0120_up_fwd	TAAGGTGACCGTTCATGTCAAAGGAGAAAAGACAGAGG	Δ*sp_0120*	This work
sp_0120_up_rev	AAA*GGCCGGCC*CTAGCGGCCAAGGAAGCCTCAACC	Δ*sp_0120*	*Fse*I; this work
sp_0120_down_fwd	AAA*GGCGCGCC*ATCAATCCAGAAACCATCGGCCAAGCC	Δ*sp_0119*/Δ*sp_0119-sp_0120*	*Asc*I; this work
sp_0120_down_rev	CAGCTCGTATCGCAGGTCATTTCAACGC	Δ*sp_0119*/Δ*sp_0119-sp_0120*	This work
ermR_fwd	*GGCGCGCC*CCGGGCCCAAAATTTGTTTGAT	Amplification of erm^R	*Asc*I; [2]
ermR_rev	*GGCCGGCC*AGTCGGCAGCGACTCATAGAAT	Amplification of erm^R	*Fse*I; [2]
mRNAcon_fwd	TAAGGTGACCGTTCATGTCAAAGGAGAAAAGACAGAGG	Test for DNA contamination in RNA extracts (>200 nt)	This work
mRNAcon_rev	CAGCTCGTATCGCAGGTCATTTCA	Test for DNA	This work

	ACGC	contamination in RNA extracts (>200 nt)	
sRNAcon_fwd	TGCTGGTATTGCCCAAGTTCCTTATGTGG	Test for DNA contamination in RNA extracts (≤200 nt)	This work
sRNAcon_rev	CCGACACTAGACCCCATGTTTGACGG	Test for DNA contamination in RNA extracts (≤200 nt)	This work

[a] Restriction sites are shown italicized and are underlined.

Supplementary Methods

Isolation and enrichment of mRNA.

At each of the assessed time-points (0, 5, 15, 60, 120, 180 min), 5 ml culture of Carolacton-treated and control cultures were taken from two duplicate cultures (100 ml) of *S. pneumoniae* TIGR4, diluted in an equal volume RNAprotect Bacterial Reagent (Qiagen), incubated at RT for 5 min, harvested by centrifugation and the resulting pellet stored at -80°C. Total RNA from was isolated using the miRNeasy mini kit (Qiagen) according to the manufacturer's instructions for separation of small RNA-enriched fractions (≤200 nt) and larger transcripts (>200 nt). Briefly, cells were washed with RNase-free water, resuspended in QIAzol lysis reagent and homogenized by vigorous vortexing for 3 min after addition of 50 mg acid-washed glass beads (diameter 100 µm). After phase separation by addition of chloroform, the aqueous phase was mixed with an equal volume of 70% (v/v) ethanol and loaded onto an RNeasy mini spin column. The flow-through was then used for enrichment of small RNAs with the RNeasy MiniElute kit (Qiagen).

The fraction containing large transcripts was isolated from the remaining RNeasy mini spin column. DNA contaminations within these fractions were removed by addition of 27 Kunitz units DNase I (RNase-Free DNase Set, Qiagen) per sample and incubation at RT for 45 min. Purification of the DNase I digestion mix was achieved with RNeasy mini spin columns (Qiagen), using the standard purification procedure described in the manual. Absence of DNA was confirmed by PCR with the primer pairs mRNAcon_fwd/mRNAcon_rev, or sRNAcon_fwd/sRNAcon_rev, respectively. Removal of ribosomal RNA in the >200 nt fraction was carried out with the Ribo-Zero rRNA Removal Kit for Gram-positive Bacteria (epicentre). Overall RNA integrity and absence of large cellular RNAs (ribosomal and >200 nt) in the samples containing small transcripts (≤200 nt) was confirmed by analysis with the Agilent 2100 BioAnalyzer using Agilent RNA 6000 Chips (Agilent Technologies). DNA library generation was carried out using the ScriptSeq™ v2 RNA-Seq Library Preparation Kit (epicentre) for long transcripts (>200 nt), and the TruSeq Small RNA Library Prep Kit (Illumina) for transcripts ≤200 nt. Libraries were sequenced on the Illumina HiSeq 2500 (Illumina) using 50-bp single-end sequencing.

RNA-seq data analysis.

First, trimming of Illumina sequencing adapter sequences of all obtained reads was done using fastq-mcf[3]. All sequenced libraries containing mRNAs >200 nt and mRNAs ≤200 nt/tRNAs were then individually mapped to the *S. pneumoniae* TIGR4 genome (NC_003028.3) using Rockhopper (v2.0.3)[4]. The location of genes shorter 200 nt, encoded within polycistronic transcripts longer 200 nt, was also taken into account. Information about operon structures within the TIGR4 genome was obtained from the Database of prOkaryotic OpeRons 2 (DOOR 2,[5]). For the analysis of differential expression of pneumococcal small regulatory RNAs (≤200 nt and

>200 nt), all reads were again mapped to the reference genome using Bowtie2 (v2.2.2)[6]. Afterwards, custom-made *.gff-files, containing locus and strand information of previously identified small RNAs (≤/>200 nt) in *S. pneumoniae* TIGR4[7-11] were used to count the mapped reads per small RNA via HTSeq (v0.6.0)[12]. Additionally, known small RNAs from other bacterial species that can be mapped to the TIGR4 genome by sequence similarity were obtained from the Bacterial Small Regulatory RNA Database (BSRD)[13] and included in the analysis. All analysed small RNAs are documented in Supplementary Dataset S3. Differential expression of transcripts was then calculated using the bioconductor edgeR (v3.1) package for R (v3.10.0)[14]. For differential analysis, the raw read counts obtained by Rockhopper or HTSeq were used. EdgeR utilizes negative binomial distribution for calculation of differential expression and is therefore considered most accurate when it comes to modelling variations in read counts between few biological replicates only[15]. False discovery rate (FDR)-adjusted P values were calculated according to[16]. FDR values of <0.01 were considered significant. For visualization of these results heat maps were generated, taking into account only genes that, in addition to a significant FDR, showed a log_2-fold change (log_2FC) of transcription of ≥±2 (or ≥±0.8 for small RNAs) at least at one point during a time course. For this, the log_2-fold values of transcript abundance obtained in edgeR were used in combination with the heatmap.2 function of the R package gplots (v2.15.0)[17]. Raw and processed RNA-seq data have been deposited in NCBI's Gene Expression Omnibus (GEO) database[18] and are accessible through GEO Series accession number GSE76979.

Flow cytometric analysis of fluorescently-labelled *S. pneumoniae* cells.

Fluorescently stained cells were analysed in triplicate on a FACSCanto flow cytometer (BD Biosciences). $DiOC_2(3)$ was excited with a blue laser at 488 nm. $DiOC_2(3)$-emitted green fluorescence (FL1) was detected through a 530 nm, 30 nm bandwidth band-pass filter, and its red

fluorescence (FL2) was detected by using a 570 nm long-pass dichroic filter and a 610 nm, 20 nm bandwidth band-pass filter. The trigger was set on the forward scatter (FSC) channel. Changes in the membrane potential were calculated using a cell size independent ratiometric technique as described previously[19]. Excitation of Syto 9/PI-stained cells was carried out at 488 nm. Green fluorescence emitted by Syto 9 (FL1) was detected through a 530 nm, 30 nm bandwidth band-pass filter. Red fluorescence emitted by PI (FL3) was detected through a 635 nm long-pass dichroic filter and a 670 nm long-pass filter. The trigger was set for the green fluorescence channel FL1. Identification of live and dead bacterial populations and quantification were carried out as described in the manufacturer's manual for the *Bac*Light Bacterial Viability Kit. A standard curve of Syto 9/PI-stained cells was prepared by mixing heat-killed and exponentially growing cells at different proportions before staining. Samples were always analysed immediately after staining. Before conducting the measurements, the instrument settings were optimized by using unstained and single-stained controls. 100,000 events were recorded for each sample; the sample analysis rate was adjusted to less than 3,000 events/s. Results were analysed using FlowJo (v10.0.7) single cell analysis software (Tree Star Inc., Ashland, USA) and the free analysis software Flowing Software (v2.5.1) (created by Perttu Terho, Cell Imaging Core, Turku Centre for Biotechnology, Finland, http://www.flowingsoftware.com/).

References.

1 Reck, M. *et al.* The biofilm inhibitor carolacton disturbs membrane integrity and cell division of *Streptococcus mutans* through the serine/threonine protein kinase PknB. *J Bacteriol* **193**, 5692-5706, doi:10.1128/JB.05424-11 (2011).

2 Lévesque, C. M. *et al.* Involvement of Sortase Anchoring of Cell Wall Proteins in Biofilm Formation by *Streptococcus mutans*. *Infect. Immun* **73**, 3773-3777, doi:10.1128/IAI.73.6.3773-3777.2005 (2005).

3 Aronesty, E. Comparison of sequencing utility programs. *Open Bioinforma J* **7**, 1-8, doi: 10.2174/1875036201307010001 (2013).

4 Tjaden, B. De novo assembly of bacterial transcriptomes from RNA-seq data. *Genome Biology* **16**, doi:10.1186/s13059-014-0572-2 (2015).

5 Mao, X. *et al.* DOOR 2.0: presenting operons and their functions through dynamic and integrated views. *Nucleic Acids Res* **42**, 654-659, doi:10.1093/nar/gkt1048 (2014).

6 Langmead, B. & Salzberg, S. L. Fast gapped-read alignment with Bowtie 2. *Nat Methods* **9**, 357-359, doi:10.1038/nmeth.1923 (2012).

7 Acebo, P., Martin-Galiano, A. J., Navarro, S., Zaballos, A. & Amblar, M. Identification of 88 regulatory small RNAs in the TIGR4 strain of the human pathogen *Streptococcus pneumoniae*. *RNA* **18**, 530-546, doi:10.1261/rna.027359.111 (2012).

8 Halfmann, A., Kovacs, M., Hakenbeck, R. & Bruckner, R. Identification of the genes directly controlled by the response regulator CiaR in *Streptococcus pneumoniae*: five out of 15 promoters drive expression of small non-coding RNAs. *Mol Microbiol* **66**, 110-126, doi:10.1111/j.1365-2958.2007.05900.x (2007).

9 Kumar, R. *et al.* Identification of novel non-coding small RNAs from *Streptococcus pneumoniae* TIGR4 using high-resolution genome tiling arrays. *BMC Genomics* **11**, 350, doi:10.1186/1471-2164-11-350 (2010).

10 Mann, B. *et al.* Control of virulence by small RNAs in *Streptococcus pneumoniae*. *PLoS Pathog* **8**, e1002788, doi:10.1371/journal.ppat.1002788 (2012).

11 Livny, J., Brencic, A., Lory, S. & Waldor, M. K. Identification of 17 *Pseudomonas aeruginosa* sRNAs and prediction of sRNA-encoding genes in 10 diverse pathogens using the bioinformatic tool sRNAPredict2. *Nucleic Acids Res* **34**, 3484-3493, doi:10.1093/nar/gkl453 (2006).

12 Anders, S., Pyl, P. T. & Huber, W. HTSeq--a Python framework to work with high-throughput sequencing data. *Bioinformatics* **31**, 166-169, doi:10.1093/bioinformatics/btu638 (2015).

13 Li, L. *et al.* BSRD: a repository for bacterial small regulatory RNA. *Nucleic Acids Res* **41**, 233-238, doi:10.1093/nar/gks1264 (2013).

14 Robinson, M. D., McCarthy, D. J. & Smyth, G. K. edgeR: a Bioconductor package for differential expression analysis of digital gene expression data. *Bioinformatics* **26**, 139-140, doi:10.1093/bioinformatics/btp616 (2010).

15 McCarthy, D. J., Chen, Y. & Smyth, G. K. Differential expression analysis of multifactor RNA-Seq experiments with respect to biological variation. *Nucleic Acids Res* **40**, 4288-4297, doi:10.1093/nar/gks042 (2012).

16 Benjamini, Y. & Hochberg, Y. Controlling the False Discovery Rate: A Practical and Powerful Approach to Multiple Testing. *Journal of the Royal Statistical Society. Series B (Methodological)* **57**, 289-300, doi:10.2307/2346101 (1995).

17 Warnes, G. R. *et al.* gplots: Various R programming tools for plotting data. R package version 2.12. 1 (2013).

18 Edgar, R., Domrachev, M. & Lash, A. E. Gene Expression Omnibus: NCBI gene expression and hybridization array data repository. *Nucleic Acids Res* **30**, 207-210, doi:10.1093/nar/30.1.207 (2002).

19 Novo, D., Perlmutter, N. G., Hunt, R. H. & Shapiro, H. M. Accurate flow cytometric membrane potential measurement in bacteria using diethyloxacarbocyanine and a ratiometric technique. *Cytometry* **35**, 55-63, doi:10.1002/(sici)1097-0320(19990101)35:1<55::aid-cyto8>3.0.co;2-2 (1999).
